# Twisted Nonlinear Optics in Monolayer van der Waals Crystals

**DOI:** 10.1021/acsnano.5c06908

**Published:** 2025-08-06

**Authors:** Tenzin Norden, Luis M. Martinez, Nehan Tarefder, Kevin W. C. Kwock, Luke M. McClintock, Nicholas Olsen, Luke N. Holtzman, June Ho Yeo, Liuyan Zhao, Xiaoyang Zhu, James C. Hone, Jinkyoung Yoo, Jian-Xin Zhu, P. James Schuck, Antoinette J. Taylor, Rohit P. Prasankumar, Wilton J. M. Kort-Kamp, Prashant Padmanabhan

**Affiliations:** † Center for Integrated Nanotechnologies, 5112 Los Alamos National Laboratory, Los Alamos, New Mexico 87545, United States; ‡ Department of Electrical Engineering, 5798 Columbia University, New York, New York 10027, United States; § Department of Chemistry, 5798 Columbia University, New York, New York 10027, United States; ∥ Department of Applied Physics and Applied Mathematics, 5798 Columbia University, New York, New York 10027, United States; ⊥ Department of Physics, 1259 University of Michigan, Ann Arbor, Michigan 48109, United States; # Department of Mechanical Engineering, 5798 Columbia University, New York, New York 10027, United States; ∇ Theoretical Division, 5112 Los Alamos National Laboratory, Los Alamos, New Mexico 87545, United States; ○ Deep Science Fund, 124345 Intellectual Ventures, Bellevue, Washington 98005, United States

**Keywords:** orbital angular momentum, vortex beams, nonlinear optics, two-dimensional semiconductors, difference frequency generation, sum frequency generation, four-wave mixing

## Abstract

In addition to a plethora of emergent phenomena, the spatial topology of optical vortices enables an array of applications in optical communications and quantum information science. Multibeam nonlinear optical processes, augmented by optical vortices, are essential in this context, providing robust access to an infinitely large set of quantum states associated with the orbital angular momentum of light. Here, we push the boundaries of vortex nonlinear optics to the ultimate limits of material dimensionality. By exploiting multipulse difference frequency, sum frequency, and four-wave mixing in monolayer quantum materials, we demonstrate their ability to independently control the orbital angular momentum and radial distribution of vortex light-fields in addition to their wavelength. Due to the atomically thin nature of the host crystal, this control spans a broad spectral bandwidth in a highly integrable platform that is unconstrained by the traditional limits of bulk nonlinear optical materials. Our work heralds an innovative path for ultracompact and scalable hybrid nanophotonic technologies empowered by twisted nonlinear light–matter interactions in van der Waals nanomaterials.

## Graphical abstract

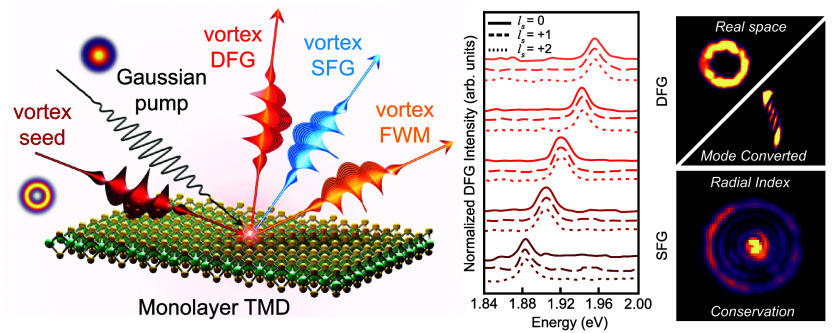

Light carries energy and momentum, the latter comprising both linear and angular components. Circularly polarized light possesses nonzero spin angular momentum (SAM), a property that has been exploited to study a wide array of material phenomena, including valley polarization,^1,2^ magnetism,^3–5^ and topology.^6,7^ Nevertheless, SAM is intrinsically restricted to a two-parameter space defined by the handedness of the light-field’s polarization. In contrast, spatially structured vortex beams, possessing helical or “twisted” wavefronts, can carry nonzero orbital angular momentum (OAM), equivalent to integer multiples of the elementary unit *ℏ*.^8–10^ OAM is therefore associated with an unbounded number of orthogonal states, indexed by the degree of wavefront twisting through a parameter known as topological charge ( l ). In addition to potentially mediating complex light–matter interactions,^11–16^ vortex beams are highly advantageous for applications that can leverage their infinite-dimensional space, including multiplexed optical communications^17–20^ and robust quantum communication paradigms.^21–24^

Most efforts aimed at exploiting vortex beams are predicated on the ability to precisely tune the wavelength and OAM of a light-field.^25^ Nonlinear optics offers a powerful technique for manipulating both of these parameters via frequency mixing processes.^26–29^ Yet, most existing approaches rely on birefringent phase matching in bulk nonlinear optical (NLO) crystals.^30^ This results in narrow conversion bandwidths defined by the material’s intrinsic nonlinear susceptibility, geometry, and relative orientation with the incident light-fields. In addition, macroscopic crystal thicknesses are required when using such materials, leading to beam aberrations that stem from volumetric effects such as spatial walk-off. Moreover, it makes such systems inherently ill-suited to integration with nanophotonic technologies (e.g., metasurfaces^31–34^ ) for free-space vortex beam generation, which have attracted significant recent attention as a vehicle to reduce the size, weight, and power consumption of OAM-based optical hardware. This is a significant hurdle given that these ultracompact devices rely on static subwavelength features and geometries. In the absence of additional functionalization (e.g., via integration with NLO crystals), this rigidity constrains their operational bandwidths and complicates their support for robust OAM tuning.

The challenges associated with conventional nonlinear crystals from the perspective of both intrinsic properties and two-dimensional (2D) device compatibility have motivated the photonics community to explore van der Waals (vdW) materials as a platform to rediscover established NLO processes at the nanoscale.^35–40^ These systems possess giant nonlinear susceptibilities and are free of adverse volumetric effects at the few-atomic-layer length scale. Moreover, they are readily compatible with complementary metal–oxide–semiconductor processes^41^ and, importantly, allow for scalable, bond-free integration with planar photonic devices via stacking or large-area growth, significantly enhancing their overall functionality.^42,43^ Nevertheless, efforts aimed at realizing broadband optical wavelength tuning via NLO processes in 2D vdW materials have entirely focused on Gaussian beams (where l = 0), leaving untapped their potential as nonlinear nanomaterials for tunable vortex light. The incorporation of the topological charge degree of freedom into vdW nonlinear optics would therefore allow us to dramatically expand the utility and shrink the length scale of an array of OAM-enabled technologies.

Here, we show that vdW quantum materials enable the on-demand, nanoscale control of the fundamental properties of optical vortices via second- and third-order NLO processes.^44^ Using monolayer transition metal dichalcogenides (TMDs), we demonstrate independent manipulation of the topological charge, radial profile, and wavelength of twisted light-fields through difference frequency generation (DFG), sum frequency generation (SFG), and four-wave mixing (FWM) (Figure 1a). As these nonlinear phenomena are supported in a single atomic layer, they are free from dispersion-induced phase mismatch, allowing for broadband conversion that further benefits from the intrinsically large optical nonlinearities of these monolayer crystals. Taken together, our work fundamentally widens the scope of vdW nonlinear optics to include the spatial degree of freedom of light, demonstrating the potential and versatility of these atomically thin systems to enable robust OAM beam tuning at the nanoscale. This, in turn, opens the door to a new paradigm for vdW-based nanophotonic technologies that can leverage the infinite dimensionality of vortex light.

**Figure 1:**
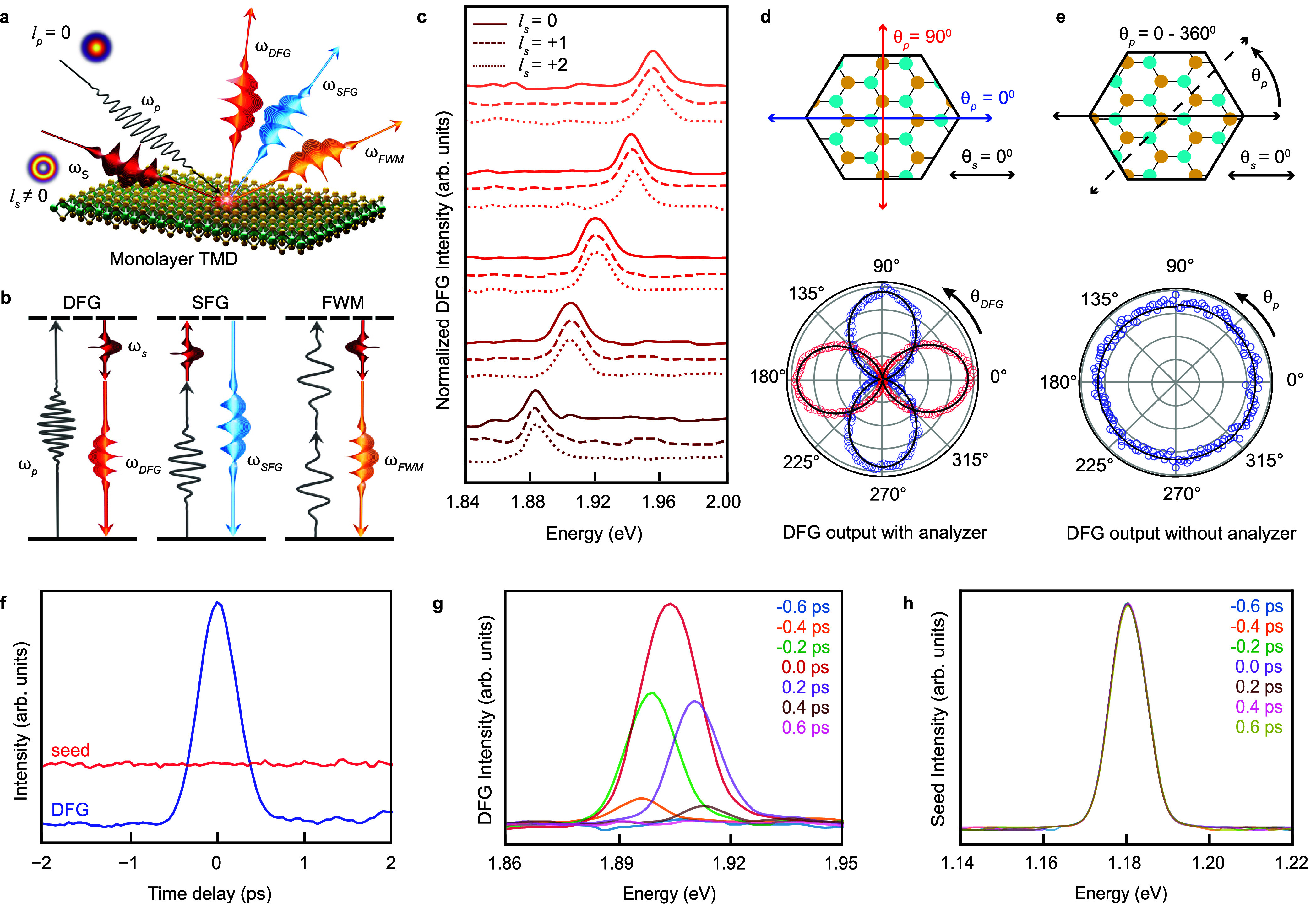
Vortex DFG from monolayer MoS_2_. (a) Schematic of NLO frequency mixing processes involving a Gaussian pump (ω_p_, l _p_ = 0) and a vortex seed (ω_s_, l _s_ ≠ 0). (b) Energy-level diagram for the generation of the optical vortex DFG (ω_DFG_), SFG (ω_SFG_), and FWM (ω_FWM_) outputs, depicting the conservation of OAM. (c) Broadband spectrum of the DFG output (ℏω_DFG_ ∼ 1.88–1.96 eV) generated by mixing a Gaussian pump (ℏω_p_^DFG^ = 3.10 eV) and a vortex seed (ℏω_s_ = 1.13–1.23 eV) for l _s_ = 0, + 1, + 2 (solid, dashed, and dotted lines, respectively). (d) Schematic (top panel) of the polarization angles of the vortex seed (θ_s_ = 0°, black arrow) and the pump (θ_p_ = 0° and 90°, blue and red arrows, respectively) with respect to the armchair crystallographic axis (i.e., the *x*-axis of the schematic) of an MoS_2_ monolayer. Polar plot (bottom panel) of the DFG output intensity as a function of the analyzer angle (θ_DFG_) when the vortex seed ( l _s_ = +1) and the Gaussian pump polarizations were either collinear (θ_s_ = θ_p_ = 0°, blue pattern) or cross-polarized (θ_s_ = 0°, θ_p_ = 90°, red pattern). The black curves are fits using a cos^2^(θ_DFG_ + ϕ) function, where θ_DFG_ = π/2 – θ_s_ – θ_p_ and ϕ is an offset angle. (e) Polar plot (bottom panel) of the DFG output intensity measured without an analyzer and plotted as a function of the Gaussian pump polarization, as depicted in the schematic (top panel), for l _s_ = +1. (f) Time-dependent trace of the DFG output (blue trace) and the reflected vortex seed (red trace) intensity measured by scanning the time delay between the Gaussian pump and vortex seed ( l _s_ = +1) pulses (seed trace offset added for clarity). (g) DFG spectrum as a function of the time delay between the Gaussian pump and vortex seed ( l _s_ = +1) pulses. (h) Vortex seed ( l _s_ = +1) spectrum as a function of the time delay between the Gaussian pump and vortex seed pulses.

## Results/Discussion

### Experimental Scheme

In all of our experiments, we utilize monolayer flakes exfoliated from bulk 2H single crystals of the prototypical vdW materials MoS_2_ and WSe_2_. The pump beam impinging on these flakes was always a standard Gaussian (i.e., with topological charge l _p_^DFG^ = l _p_^SFG^ = l _p_^FWM^ = l _p_ = 0). The pump energies used for the DFG, SFG, and FWM studies were ℏω_p_^DFG^ = 3.10 eV, ℏω_p_^SFG^ = 1.63 eV, and ℏω_p_^FWM^ = 1.54 eV, respectively. The seed beam was a Laguerre–Gaussian vortex, which we imparted with various nonzero topological charge values (except in control experiments where it was deliberately set to 0, yielding a standard Gaussian seed beam). The seed photon energy was tuned over the same range (i.e., 1.13–1.23 eV) for all three NLO processes under investigation. Hereafter, for simplicity, we use the labels ℏω_s_ and l _s_ for the seed’s photon energy and topological charge, respectively, for all studies.

### Manipulating Vortex Pulses through Difference Frequency Mixing in a vdW Monolayer

DFG is a second-order nonlinear frequency downconversion process that is integral to optical parametric amplification,^45^ which forms the basis of modern tunable coherent light sources. Here, the interaction of a high-energy pump photon and a low-energy seed photon leads to the generation of a photon at their energy difference (i.e., ℏω_DFG_ = ℏω_p_^DFG^ – ℏω_s_, left panel of Figure 1b). Figure 1c shows the spectrum of the DFG output from a monolayer of MoS_2_ generated using a Gaussian pump (ℏω_p_^DFG^ = 3.10 eV, l _p_ = 0) and various seed photon energies (ℏω_s_ = 1.13–1.23 eV) at different values of l _s_. It is immediately evident that the vdW crystal supports broad-spectrum frequency conversion.^35^ More importantly, the spectrum of the DFG output is insensitive to the value of l _s_, providing compelling evidence that wavelength and topological charge conversion are decoupled processes. We note that the efficiency of the observed vortex DFG is comparable to what was recently reported in the literature for the analogous all-Gaussian-beam process^35^ (see Figure S9). In both cases, however, the efficiency cannot be effectively scaled by using thicker flakes. 2H-stacked TMD multilayer flakes exhibit layer-dependent inversion symmetry breaking; odd-layered flakes break inversion symmetry, whereas even-layered flakes preserve inversion symmetry. As such, the conversion efficiency of second-order NLO processes becomes vanishingly small for even-layered crystals and drops precipitously for odd-layered crystals as the layer number is increased.^46^ However, utilizing vdW materials with intrinsically larger second-order susceptibilities (compared to TMDs) and broken inversion symmetry that is independent of layer number, such as NbOCl_2_, can allow us to potentially circumvent this issue. Indeed, the absolute nonlinear efficiency of such materials generally scales with layer number.^36^ This allows us to use multilayer flakes, still significantly thinner than the pump, seed, or NLO output wavelengths, to yield dramatic enhancements of vortex DFG efficiency across a broad photon energy range (see Figure S9).

The polarization properties of frequency-mixed outputs resulting from NLO processes are uniquely associated with the point group symmetry of the crystal. Theoretical analysis (see the Supporting Information section VIII) yields an expected polarization of θ_DFG_ = π/2 – θ_s_ – θ_p_ for the DFG output from monolayer TMDs, given their *D*_3*h*_ point group symmetry. Here, θ_s_, θ_p_, and θ_DFG_ are the seed, pump, and DFG output linear polarization angles with respect to the crystal’s armchair direction, respectively. To confirm this dependence experimentally, we performed polarimetry measurements, in turn allowing us to verify that the vortex DFG output originated entirely from the MoS_2_ monolayer. Figure 1d shows the polarization dependence of the vortex DFG output intensity for two different configurations of the relative angle between the Gaussian pump and vortex seed ( l _s_ = +1) polarizations. This was measured by rotating an analyzer placed before the detector. As shown in the top panel schematic of Figure 1d, the seed polarization angle was fixed to the crystal’s armchair direction (θ_s_ = 0°), and the pump polarization was either collinear (θ_p_ = 0°, corresponding to the blue pattern in the bottom panel of Figure 1d) or orthogonal (θ_p_ = 90°, corresponding to the red pattern in the bottom panel of Figure 1d) to this axis. The bilobed structure and the π/2 shift in the orientations of the two patterns match our theoretical expectations based on the crystal’s symmetry. Additionally, the intensity of the DFG output measured without an analyzer does not show any dependence on the relative polarization angles of the pump and the seed^35^ (bottom panel of Figure 1e). Similar analyses for seed pulses with other values of l _s_ show identical results for both MoS_2_ and WSe_2_ monolayers (see Figures S3 and S5). This indicates the lack of appreciable impact of the seed’s topological charge on the polarization properties of the DFG output, highlighting their relative independence in the vortex DFG process.

An analysis of the time-domain dynamics of the vortex DFG output intensity, relative to the delay between the Gaussian pump and vortex seed ( l _s_ = +1) pulses, reveals a Gaussian temporal profile (blue trace in Figure 1f) associated with a time-dependent buildup of the DFG spectrum (Figure 1g). This implies that the DFG process occurs only when the seed and pump pulses are spatiotemporally overlapped. In contrast, the reflected seed shows no appreciable change in either intensity (red trace in Figure 1f) or spectrum (Figure 1h) over the time frame of the DFG process. The lack of measurable seed enhancement, consistent with previous reports of all-Gaussian-beam processes,^35^ indicates that parametric amplification is either absent or exceptionally weak in our studies. This is likely due to our low pump-to-seed intensity ratios,^45^ stemming from the need to mitigate sample damage while ensuring a seed with an intensity above our detection threshold and spectral range within the operational regime of the spatial light modulator (SLM).

We then directly imaged the seed and DFG beams to characterize their OAM state. Figure 2a shows the real-space seed beam images for l _s_ = 0–6, revealing a characteristic annular intensity profile for l _s_ ≠ 0 and diameter that is correlated to the magnitude of the topological charge (i.e., a larger | l _s_| leads to a seed with a larger geometric radius). Astigmatic mode conversion, introduced by a cylindrical lens, allowed us to convert the vortex beam into a characteristic intensity pattern at the focal plane, where the number and skew of the resulting fringes are directly related to the beam’s OAM magnitude and sign, respectively, enabling quantification of its topological charge.^47^ Here, the number of bright fringes in the seed, *N*_F_^s^, is equivalent to | l _s_| + 1, as confirmed in the images shown in Figure 2b. Intriguingly, the real space images of the DFG output show that it inherits an annular intensity profile when l _s_ ≠ 0, with its diameter being approximately equivalent to that of the seed for any given value of l _s_ (Figure 2d). Moreover, mode conversion of the DFG output reveals the presence of fringes with *N*_F_^DFG^ = *N*_F_^s^ but with skew directions that are opposite to that of the seed (Figure 2c). Taken together, this indicates a conservation of the magnitude but an inversion of the sign of the topological charge (i.e., *l*_DFG_ = −*l*_s_) in the vortex DFG process.

**Figure 2:**
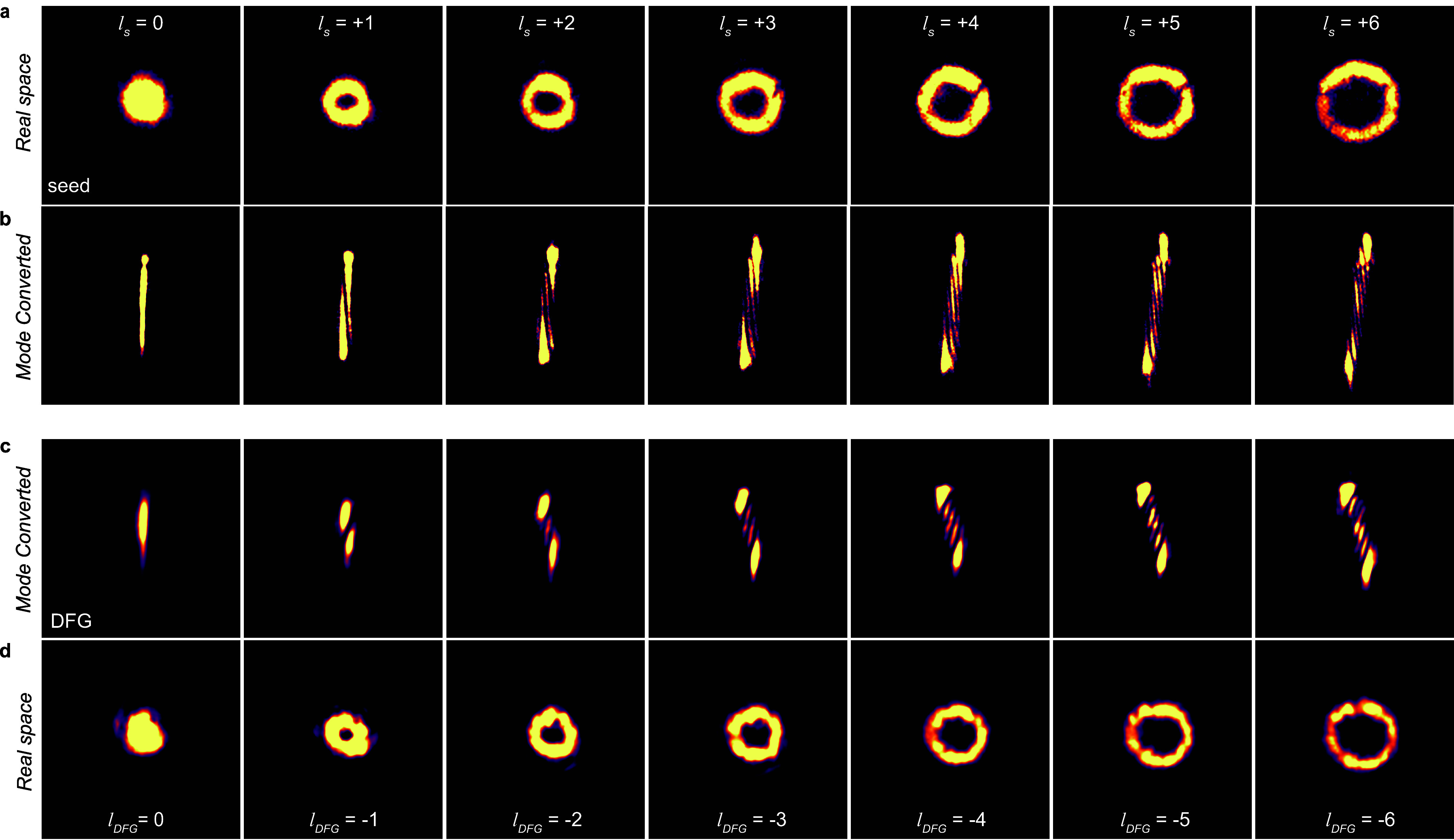
Intensity profile images of vortex DFG from monolayer MoS_2_. (a) Real space images of the intensity profile of the seed beam, ℏω_s_ = 1.18 eV, for seed topological charges l _s_ = 0–6. (b) Mode converted images of the seed at the focal plane of a cylindrical lens (*f* = 120 mm). The number of fringes, *N*_F_^s^, is equivalent to | l _s_| + 1, and the direction of the skew corresponds to the sign of l _s_ (i.e., right to left skew from top to bottom corresponds to positive values). (c) DFG output (ℏω_DFG_ = 1.92 eV) mode converted images showing the number of fringes *N*_F_^DFG^ = *N*_F_^s^ but with opposite skew directions (i.e., left to right from top to bottom), reflecting the fact that $l_{DFG\,}\,={-l}_{s}.$ (d) Corresponding real space images of the DFG output. All data were taken with a Gaussian pump (ℏω_p_^DFG^ = 3.10 eV, l _p_ = 0). Image intensities were made comparable by adjusting the acquisition settings of the EMCCD.

To contextualize our observations, we theoretically consider the case of a monolayer NLO crystal on an inversion symmetric substrate illuminated by a structured optical field. The incident light is modeled as the superposition of *N* monochromatic waves with frequencies {ω_1_, ω_2_, ···, ω_*N*_}. This allows for the derivation of the reflected frequency-mixed electric field as

$$E_{R}(R,\omega )=2\pi \hbar \sum_{m=1}\sum_{\begin{array}{c} \left(\zeta_{1},\zeta_{2}\cdots \zeta_{m}\right)\\ \in \{\pm \omega_{j}\} \end{array}}R^{\left(m\right)}(\{\zeta_{j}\}):E_{0}^{\zeta_{1}}(R)\cdot \cdot \cdot E_{0}^{\zeta_{m}}(R)\delta \left(\hbar \omega -\sum_{j=1}^{m}\hbar \zeta_{j}\right)$$ 1

where *m* is the harmonic order, $R^{\left(m\right)}$ is the generalized Fresnel reflection coefficient tensor, and $E_{0}^{\zeta }$ is the vectorial spatial profile of the incident field oscillating at frequency ζ ∈ {± ω_1_, ± ω_2_, ···, ± ω_*N*_}. This expression is valid for arbitrary nonlinear orders and for any structured light-fields within the paraxial approximation while explicitly accounting for the 2D nature of the quantum material (see Methods/Experimental Section).

Equation 1 allows us to draw several conclusions regarding vortex NLO processes involving 2D materials. First, for the case where there are only two incident fields (i.e., a pump and a seed) with energy ℏω_p_ and ℏω_s_ (i.e., $\zeta_{j}=\left\{\omega_{p}.\pm \omega_{s}\right\}$ ), the Dirac delta in eq 1 enforces energy conservation,

$$\hbar \omega_{\mathrm{out}}=\hbar |\alpha \omega_{p}+\beta \omega_{s}|$$ 2

Here, −*m* ≤ α,β ≤ *m* are integers corresponding to the difference between the number of positive and negative ω_p_ and ω_s_ field components contributing to the nonlinear process. For the case of DFG, eq 2 reduces to the observed ℏω_out_ = *ℏ* |ω_p_ – ω_s_|. Moreover, eq 1 does not impose any conditions on the linear momentum of the input or reflected fields. This implies that 2D vdW crystals enable broadband frequency conversion for all multibeam frequency mixing processes, regardless of the input fields’ spatial profile or OAM, in agreement with the OAM-agnostic DFG tuning bandwidth seen in Figure 1c. In addition, material properties and symmetries are entirely encoded in $R^{\left(m\right)}$ , allowing them to modulate the light-fields’ polarization degrees of freedom (and therefore SAM), but not their spatial structure or OAM, as seen from the invariance of the DFG polarimetry results for various values of l _s_ (see Figure S3). This asymmetry between the coupling of material degrees of freedom to OAM and SAM is also at the heart of the complexity in driving electronic currents and transferring OAM from weakly focused beams^11^ to charge carriers in solids. Lastly, when the incident fields are eigenmodes of the paraxial wave equation with well-defined topological charge l _*j*_ (i.e., $E_{0}^{\zeta_{j}}\propto e^{\pm il_{j}\phi }$ ), eq 1 implies that the OAM of the output fields, due to an *m* ^th^ order nonlinear process, is determined by the sum of the OAM of all contributing input beams. For the case of a pump and seed with topological charges l _p_ and l _s_, respectively,

$$l_{\mathrm{out}}=\alpha l_{p}+\beta l_{s}$$ 3

It is evident that the frequency-mixed field inherits the OAM of the constituent inputs (e.g., α, β = +1, −1 for DFG), consistent with the results presented in Figure 2.

### SFG and Radial Mode Matching

SFG is the second-order counterpart to DFG and involves the frequency upconversion of two incident photons, with potentially different photon energies, producing a photon with energy ℏω_SFG_ = ℏω_p_^SFG^ + ℏω_s_ (middle panel of Figure 1b). To demonstrate the vortex SFG process, we performed a similar experiment on a monolayer of MoS_2_, utilizing a Gaussian pump (ℏω_p_^SFG^ = 1.63 eV, l _p_ = 0, Figure 3a) and a vortex seed (ℏω_s_ = 1.18 eV, l _s_ = +3, Figure 3b). As seen in Figure 3c, the SFG output again inherits the annular intensity profile of the seed. However, in contrast to the DFG case, though *N*_F_^SFG^ = *N*_F_^s^, the mode converted seed and SFG images (bottom panels of Figure 3b,c, respectively) are skewed in the same direction. This confirms the equivalence of both the magnitude and sign of the topological charge of the two beams, which can be understood from eq 3, with α = β = +1. Similar results were obtained for the other values of l _s_ (see Figure S6), in addition to characterizations of the broad wavelength tunability and polarization of the SFG responses (matching theoretical expectations vis-à-vis crystal symmetry; see the Supporting Information section VIII), which were both insensitive to the seed’s topological charge (see Figure S7). The efficiency of the vortex SFG process in TMD monolayers is very similar to that of vortex DFG, and once again, significant enhancements can be achieved by using vdW crystals with larger nonlinearities and advantageous symmetry (see Figure S9).

**Figure 3:**
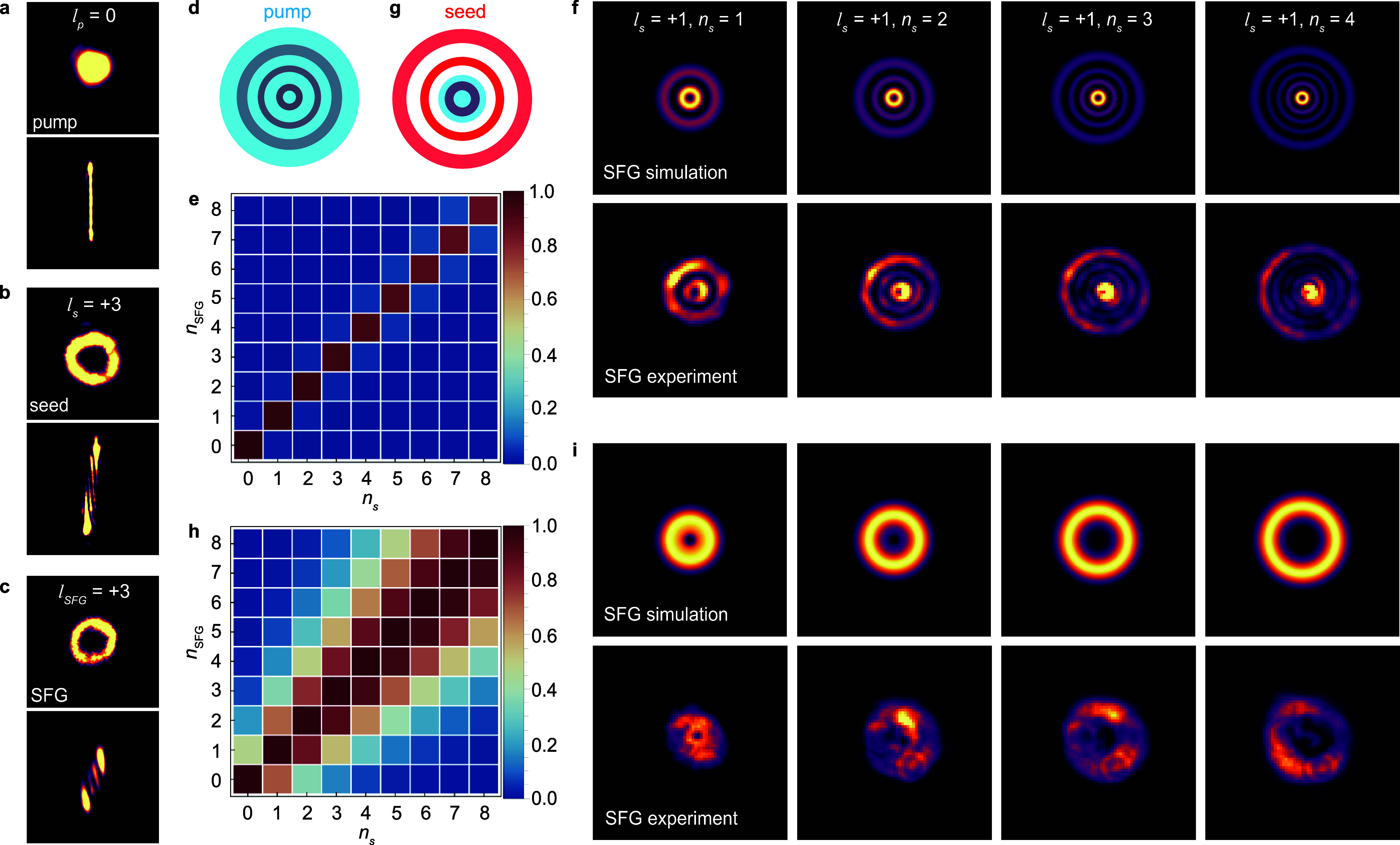
SFG and radial index manipulation from monolayer MoS_2_ with vortex beams. (a–c) Real space (top panel) and mode converted (bottom panel) images of the (a) Gaussian pump (ℏω_p_^SFG^ = 1.63 eV, l _p_ = 0), (b) vortex seed (ℏω_s_ = 1.18 eV, l _s_ = +3), and (c) SFG output (ℏω_SFG_ = 2.81 eV, l _SFG_ = +3), where *N*_F_^SFG^ = *N*_F_^s^ and the mode converted patterns of the seed and SFG output have the same skew. (d) Illustration of the SFG experimental configuration with a large Gaussian pump beam waist (blue) overlapping the entire vortex seed beam (red) with nonzero radial index, *n*_s_. (e) Overlap integral (Λ) matrix showing perfect conservation of the radial index, *n*_SFG_ = *n*_s_. (f) Simulations (top panel) and experimentally observed (bottom panel) intensity profiles of the SFG output for vortex seeds with l _s_ = +1 and *n*_s_ = 1–4 for the experimental configuration associated with (d) and (e). (g) Illustration of the SFG experimental configuration where the Gaussian pump beam waist (blue) is the same as the vortex seed’s (red) central annulus. (h) Overlap integral (Λ) matrix showing the breakdown of the radial index conservation. (i) Simulated (top panel) and experimentally observed (bottom panel) SFG output intensity profiles for vortex seeds with l _s_ = +1 and *n*_s_ = 1–4 for the experimental configuration associated with (g) and (h).

Apart from topological charge, Laguerre–Gaussian vortices have another indexed spatial degree of freedom, namely, the radial index *n*; a vortex beam with *n* ≠ 0 is characterized by an intensity profile comprising *n* + 1 concentric annuli. Thus far, we have exclusively considered vortex seed pulses with *n*_s_ = 0 but now turn to processes where *n*_s_ ≠ 0. Radial mode conversion in a two-beam second-order mixing process is determined by an overlap integral involving the pump, seed, and frequency-mixed fields that are part of the nonlinear process, given by (see Methods/Experimental Section)­

$$\Lambda_{\begin{array}{c} n_{p}n_{s}n_{i}\\ l_{p}l_{s}l_{i} \end{array}}=2\pi \delta_{l_{i},l_{p}\pm l_{s}}\int_{0}^{\infty }Ru_{n_{p},l_{p}}^{\omega_{p}}(R)u_{n_{s},l_{s}}^{\omega_{s}}(R)u_{n_{i},l_{i}}^{*}(R)dR$$ 4

where $u_{n,l}^{\omega }$ (= $u_{n,l}$ , ω ≥ 0; $u_{n,l}^{*}$ , ω < 0) are the orthonormal Laguerre–Gaussian modes and the ± symbol appearing in the subscript of the Kronecker delta function enforces OAM conservation for SFG and DFG, respectively. We consider the case where the pump is a Gaussian mode (i.e., *n*_p_ = l _p_ = 0) focused on the monolayer. When the seed is a Laguerre–Gaussian vortex also focused on the monolayer, the mode functions $u_{n,l}$ are real-valued and depend on the absolute value of l . As a result, the Λ values are identical for SFG and DFG, allowing us to concentrate on the former without any loss of generality. We begin with the case where the Gaussian pump beam waist is significantly larger than that of the Laguerre–Gaussian vortex seed (see Figure 3d). The plot of Λ in Figure 3e shows nearly perfect radial mode matching in the SFG process, an effective conservation law in which *n*_SFG_ = *n*_s_. This can be clearly seen in simulations of the vortex SFG output intensity profile for a seed with l _s_ = +1 and *n*_s_ = 1–4 (top panels of Figure 3f) and is in remarkably close agreement with the experimentally observed SFG output profiles from a monolayer of MoS_2_ (bottom panels of Figure 3f). Such radial index conservation, under similar mode matching of the pump and seed, is expected for any uniform vdW monolayer with broken inversion symmetry as they identically eliminate volumetric aberrations.

A considerably different situation occurs in a geometry where the Gaussian pump beam waist is overlapped with only the central annulus of the vortex seed (Figure 3g). Here, mode conservation breaks down, giving way to a distribution of radial modes in the SFG output for any given *n*_*s*_ (Figure 3h). The result is a single diffuse annulus in the far-field, which can be seen in both the simulated (top panel of Figure 3i) and experimentally obtained (bottom panel of Figure 3i) SFG output profiles. We note that the small, Airy patternlike features in the lower panels of Figure 3i are artifacts from transmissive optics in our system. Furthermore, the overall slight variation in the image contrast of the annuli is due to the increasing difficulty in collecting the emitted nonlinear output for larger values of the radial index due to the relatively low numerical aperture of our objective.

### FWM

Thus far, we have discussed three-wave vortex NLO processes enabled by the second-order nonlinearity of monolayer crystals. However, FWM is also possible through the third-order susceptibility,^44^ χ^(3)^. For example, two pump photons and a seed photon can mix to yield an FWM output with a photon energy ℏω_FWM_ = ℏω_p1_^FWM^ + ℏω_p2_^FWM^ – ℏω_s_ (right panel of Figure 1b). FWM is important in applications such as extreme ultraviolet light generation and control,^48,49^ near-field imaging,^50^ frequency comb generation,^51^ and quantum state generation,^52^ making its nanoscale realization with vortex light of particular interest. In our experiment, we make use of two-photon excitation by the same Gaussian pump, and as such, ℏω_p1_^FWM^ = ℏω_p2_^FWM^ = ℏω_p_^FWM^= 1.54 eV; the seed photon energy ranges from ℏω_s_ = 1.13–1.23 eV. As shown in Figure 4a, the MoS_2_ monolayer supports vortex FWM over a range of seed photon energies, with nearly equivalent spectral profiles regardless of the value of l _s_.

**Figure 4:**
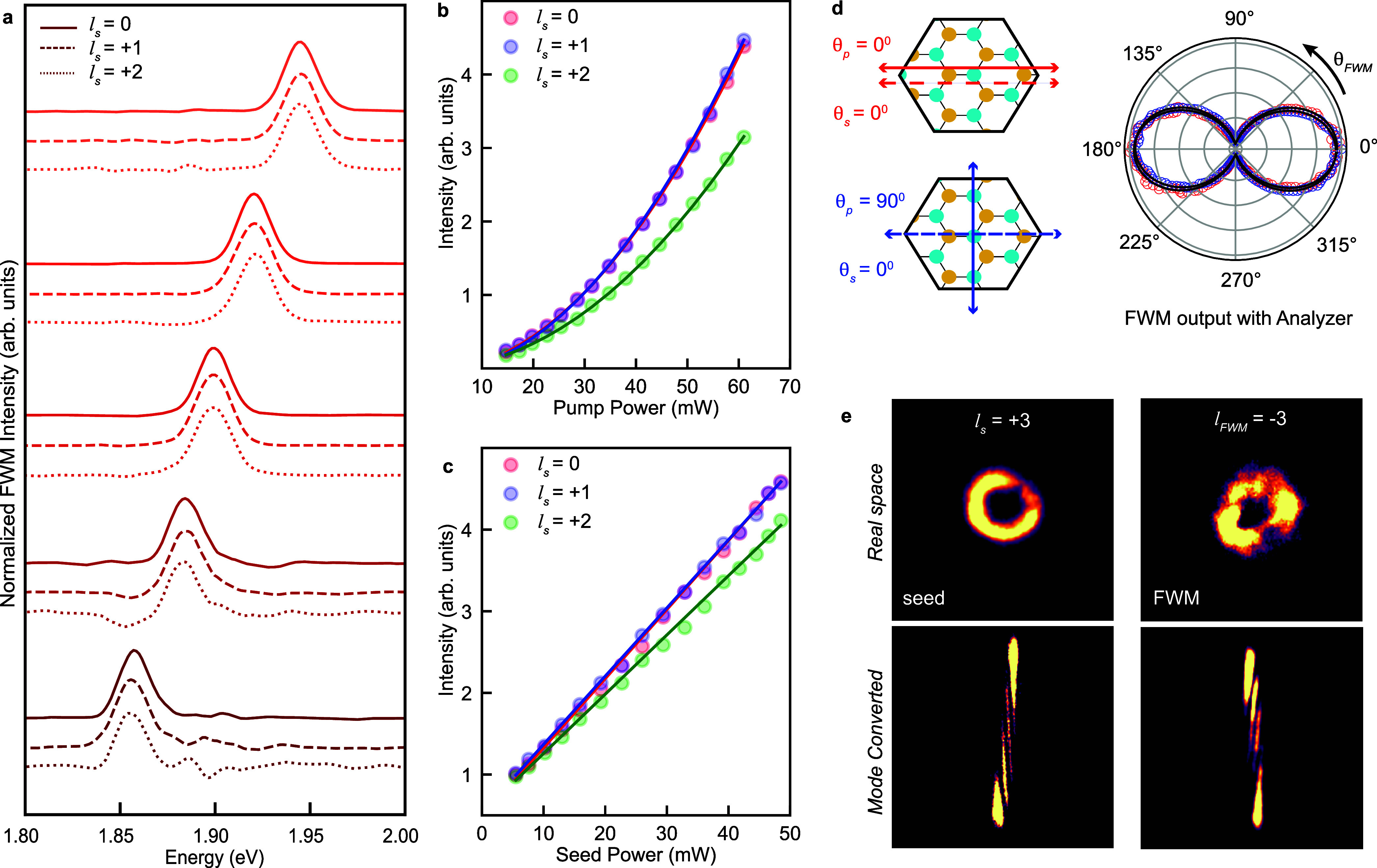
Vortex FWM from monolayer MoS_2_. (a) Broad spectrum tuning of the FWM output (ℏω_FWM_ ∼ 1.85–1.95 eV) for seed beams (ℏω_s_ = 1.13–1.23 eV) with l _s_ = 0, +1, +2 (solid, dashed, and dotted lines, respectively) and Gaussian pump beam (ℏω_p_^FWM^ = 1.54 eV, l _p_ = 0). (b) Gaussian pump power dependence of the FWM output (ℏω_FWM_ = 1.90 eV) for l _s_ = 0, + 1, + 2 (red, blue, and green circles, respectively), with solid lines representing square-law fits. (c) Seed power dependence of the FWM output (ℏω_FWM_ = 1.90 eV) for l _s_ = 0, +1, +2 (red, blue, and green circles, respectively), with solid lines representing linear fits. (d) Polar plot (right panel) of the FWM output intensity as a function of the analyzer angle (θ_FWM_) for l _s_ = +3. The schematic (left panel) shows that the vortex seed beam polarization is kept parallel to the armchair axis of the crystal (θ_s_ = 0°), while the Gaussian pump beam polarization is either parallel (θ_p_ = 0°, corresponding to the red pattern in the right panel) or perpendicular (θ_p_ = 90°, corresponding to the blue pattern in the right panel) to the armchair axis. (e) Real space annular intensity profile (top panel) and mode converted images (bottom panel) of the vortex seed (left panels) and the FWM output (right panels) for l _s_ = +3. The bottom panels show that *N*_F_^FWM^ = *N*_F_^s^ and that the patterns have opposite skews. Images were taken at ℏω_FWM_ = 1.90 eV for the FWM output and ℏω_s_ = 1.18 eV for the vortex seed.

The FWM process is driven by a nonlinear polarization source term of the form *P*_*i*_^NL^ ∝ χ_*ijko*_^(3)^ *E*_*j*_^p^ *E*_*k*_^p^ *E*_*o*_^s^, where *E* ^p^ (*E* ^s^) is the electric field of the pump (seed) and *i*, *j*, *k*, and *o* are Cartesian directions. This leads to an expected square-law dependence on the pump power and linear dependence on seed power, which we observe experimentally, as shown in Figure 4b,c, respectively. The slight drop in the intensity of the FWM output for the l _s_ = +2 case is most likely due to the decreased efficiency of the FWM process for seeds with larger geometric diameters, owing to their diminished seed intensity and mode overlap with the pump. In addition, considering the form of *P*_*i*_^NL^ for FWM, the intensity polar pattern is expected to show a bilobed shape oriented along the seed polarization direction (see the theoretical discussion in the Supporting Information section VIII), which we confirmed experimentally as shown in the right panel of Figure 4d. Finally, the top-right panel of Figure 4e shows the annular intensity pattern of the FWM output, qualitatively similar in size to that of the seed pulse (top-left panel, Figure 4e). However, the sign of the topological charge of the FWM output is inverted with respect to the seed, as seen from the opposing skews of the mode converted images of the seed (bottom-left panel, Figure 4e) and FWM output (bottom-right panel, Figure 4e). In analogy to the DFG and SFG processes, the OAM conservation law for FWM obtained from eq 3 is l _FWM_ = 2 l _p_– l _s_. Given that l _p_ = 0, we find that l _FWM_ = − l _s_ = −3 as observed experimentally. While we have discussed the case for l _s_ = +3, results for other values of l _s_ are consistent with this analysis (see Figure S8). Ultimately, this confirms that wavelength tunability and topological charge conversion are fully decoupled, even in higher-order vortex NLO processes, confirming the higher-harmonic validity of eq 1.

## Conclusions

Our results underscore the ability of monolayer vdW materials to enable the broad-spectrum manipulation of all of the fundamental properties of twisted light within an atomically thin platform. In this way, we emphasize the potential of such materials to support the scalable and passive functionalization of 2D devices for the free-space generation of OAM beams,^31–34^ paving the way toward robust, monolithic, nanometer-thin sources of broadly tunable vortex and higher-order structured light. This, in turn, could lead to significant advancements in our ability to develop vdW-based nanophotonic platforms for a multitude of applications, including high-density optical data transmission,^18–20,53^ super-resolution imaging,^54,55^ and quantum information.^21–24^ Moreover, although we have focused on DFG, SFG, and FWM, we envision that vdW monolayers can also support other exotic vortex NLO phenomena. This could further push the boundary of nanoscale vortex nonlinear optics to enable the creation of tunable twisted quantum states of light through processes such as spontaneous parametric downconversion^36^ or even extreme ultraviolet OAM states through solid-state high-harmonic generation.^56^ In addition, the highly nonuniform nature of optical vortices could enable entirely new emergent nonlinear phenomena born from higher-order multipolar light–matter interactions. We anticipate that many new opportunities will emerge at the intersection of structured light and vdW quantum nanomaterials through the harnessing of spatiotemporal light–matter interactions.

## Methods/Experimental Section

### Crystal Synthesis and Sample Preparation

Large-area monolayers of MoS_2_ were exfoliated from commercially purchased (HQ Graphene) bulk single crystals via the Au tape exfoliation method.^57^ Here, a 150 nm thin layer of Au tape was prepared by depositing Au onto a polished silicon wafer, followed by spin coating with a protective layer of polyvinylpyrrolidone (PVP). The Au tape was then removed from the silicon wafer using thermal release tape (Semiconductor Equipment Corp. Revalpha RA-95LS­(N)). The large-area monolayer flake was mechanically exfoliated by lightly pressing the Au tape onto the surface of a bulk MoS_2_ crystal. The Au tape was placed onto a 0.3 mm glass substrate, which was then heated on a hot plate at 135°C to remove the thermal release tape. Once the thermal release tape was removed, the PVP protection layer was dissolved by soaking the substrate in deionized water for 3 h and acetone for 1 h. Subsequently, the Au film was dissolved in an I_2_/KI etchant solution for 5 min. Finally, the sample was again soaked in deionized water for 2 h, before rinsing it with isopropanol and drying with N_2_ gas. The single layer character of the sample was confirmed using optical contrast and Raman spectroscopy (Figure S1a–c). The monolayer of WSe_2_ was prepared using standard mechanical exfoliation techniques from high-quality single crystals of WSe_2_ synthesized using a previously described self-flux method.^58^

### Time-Resolved Vortex NLO Spectroscopy and Imaging

NLO experiments were conducted on a time-resolved structured light microscopy system (Figure S1d). A tunable Ti:sapphire oscillator (1.15–1.82 eV, ∼150 fs, ∼80 MHz) was used to pump an optical parametric oscillator (OPO), which emitted a tunable signal in the near-infrared (0.78–1.24 eV, ∼150 fs, ∼80 MHz). In all our studies, the pump pulse supplied by the oscillator was kept as a Gaussian ( l _p_ = 0) and the seed pulse supplied by the OPO was spatially structured into a Laguerre–Gaussian optical vortex ( l _s_ ≠ 0) using a liquid crystal on silicon phase-only SLM (HOLOEYE PLUTO-2.1) with an operational range of 1.13–2.95 eV. Here, the Gaussian seed beam from the OPO was first expanded with a telescope and then converted into a vortex beam after being reflected from the SLM, which was encoded with a particular phase mask (see Figure S2), at near-normal incidence. The pump (Gaussian) and the seed (vortex) pulses were then combined collinearly using a 950 nm short-pass dichroic filter, reflected with a 50:50 nonpolarizing beam splitter, and focused onto the sample plane using a 20X (0.42 NA) infinity-corrected apochromatic objective. The pulses were spatiotemporally overlapped on the crystal by adjusting a mechanical delay line that was part of the pump beam path. The focal spot diameter of the seed on the sample ranged from 4 to 12 μm for l _s_ = 0–6. Therefore, to ensure full spatial overlap of the pump and seed on the sample, the pump’s focal spot diameter was set to ∼15 μm (except for the radial mode matching SFG studies where the pump beam was ∼22 μm to accommodate the larger geometric size of the seed with *n*_s_ ≠ 0).

For the SFG and FWM processes, the photon energy of the pump was set to ℏω_p_^SFG^ = 1.63 eV and ℏω_p_^FWM^ = 1.54 eV, respectively, while for the DFG process, the pump was frequency-doubled to ℏω_p_^DFG^ = 3.10 eV using a 1 mm thick type-I bismuth triborate crystal. The DFG, SFG, and FWM outputs were collected through the same objective in a reflection geometry and picked off using a 490 nm short-pass dichroic, 650 nm long-pass dichroic, or a 50:50 600–1700 nm beam splitter, respectively. The pump and seed beams were blocked by placing a combination of interference filters in the collection path before the detectors to isolate the NLO output of interest. A spectrometer equipped with a thermoelectrically cooled charge-coupled device (CCD) camera was used to record the spectra of the generated outputs. For the polarization- and intensity-dependent measurements, the output beam was directed into a photomultiplier tube (PMT). Here, the pump beam was modulated with an optical chopper and the PMT signal was fed to a lock-in amplifier referenced to the chopper frequency. The incident pump and signal polarization angles were carefully set independently with reference to the armchair axis of the crystal using λ/2 waveplates, and the polarization dependencies of all outputs were measured by rotating an analyzer placed before the PMT. The analyzer was also removed to measure the raw intensity of the NLO outputs under pump polarization rotation by rotating the pump’s λ/2 waveplate. All beams were also directed to a silicon electron-multiplying CCD (EMCCD) camera (Teledyne Princeton Instruments ProEM), to image either their real-space intensity profiles or their mode converted profiles (at the focus of a cylindrical lens placed before the EMCCD, *f* = 120 mm), the latter to determine the magnitude and sign of their topological charge.

### Theory of Nonlinear Vortex Light Scattering in 2D vdW Crystals

We consider the case of a monolayer crystal lying over a substrate with a linear refractive index *n*(ω) that is illuminated by monochromatic structured light-fields. The electromagnetic fields on the monolayer surface (*z* = 0) at a position ***R*** and with frequency ω satisfy^59^

$$\hat{z}\times [E_{T}(R,\omega )-E_{0}(R,\omega )-E_{R}(R,\omega )]=0$$ 5

$$\hat{z}\times [H_{T}(R,\omega )-H_{0}(R,\omega )-H_{R}(R,\omega )]=J(R,\omega )$$ 6

where ***J*** (***R***, ω) is the monolayer’s induced surface current density due to both linear and nonlinear frequency mixing processes. Also, ***E***_0,R,T_ and ***H***_0,R,T_ are the incident, reflected, and transmitted electromagnetic fields, respectively. In the following, we assume that the input beams impinge normal to the monolayer, neglect nonlinear effects due to the substrate, and consider only contributions up to leading order in the paraxial approximation (i.e., $H_{0,R}(R,\omega )≃\pm \frac{1}{\mu_{0}c}\hat{z}\times E_{0,R}(R,\omega )$ and $H_{T}(R,\omega )≃\frac{n(\omega )}{\mu_{0}c}\hat{z}\times E_{T}(R,\omega )$ ). Hence,

$$E_{T}(R,\omega )=\frac{2}{1+n(\omega )}E_{0}(R,\omega )-\frac{\mu_{0}c}{1+n(\omega )}J(R,\omega )$$ 7

$$E_{R}(R,\omega )=\frac{1-n(\omega )}{1+n(\omega )}E_{0}(R,\omega )-\frac{\mu_{0}c}{1+n(\omega )}J(R,\omega )$$ 8

To compute ***J*** (***R***, ω), we note that in the weak interaction regime the time-domain surface current density can be expanded in powers of the electromagnetic field on the surface of the monolayer as $J(R,t)=\sum_{m}J^{\left(m\right)}(R,t)$ , where^60^

$$J^{\left(m\right)}(R,t)=\frac{1}{(2\pi {)}^{3m}}\int dq_{1}\cdot \cdot \cdot dq_{m}\int d\omega_{1}^{\prime }\cdot \cdot \cdot d\omega_{m}^{\prime }e^{i\sum_{j=1}^{m}(q_{j}\cdot R-\omega_{j}^{\prime }t)}\times {\overset{↔}{\sigma }}^{\left(m\right)}(\{q_{j}\};\{\omega_{j}^{\prime }\}):E_{T}(q_{1},\omega_{1}^{\prime })\cdot \cdot \cdot E_{T}(q_{m},\omega_{m}^{\prime })$$ 9

is the *m* ^th^ order nonlinear contribution to the surface electronic current. The notation {***q***_*j*_}, {ω_*j*_′} denotes the entire set of linear momentum and frequency variables that the nonlinear conductivity tensor ${\overset{↔}{\sigma }}^{\left(m\right)}$ depends upon. In practice, however, the linear momentum per photon for propagative light-fields is significantly smaller than that of the charge carriers in the monolayer. Thus, one can neglect spatial dispersion in the nonlinear conductivity, resulting in

$$J^{\left(m\right)}(R,t)=\frac{1}{(2\pi {)}^{m}}\int d\omega_{1}^{\prime }\cdot \cdot \cdot d\omega_{m}^{\prime }e^{-i\sum_{j=1}^{m}\omega_{j}^{\prime }t}{\overset{↔}{\sigma }}^{\left(m\right)}(\{\omega_{j}^{\prime }\}):E_{T}(R,\omega_{1}^{\prime })\cdot \cdot \cdot E_{T}(R,\omega_{m}^{\prime })$$ 10

Fourier-transforming the previous equation to the frequency space,

$$J(R,\omega )=\sum_{m}\frac{1}{(2\pi {)}^{m-1}}\int d\omega_{1}^{\prime }\cdot \cdot \cdot d\omega_{m}^{\prime }\times {\overset{↔}{\sigma }}^{\left(m\right)}(\{\omega_{j}^{\prime }\}):E_{T}(R,\omega_{1}^{\prime })\cdot \cdot \cdot E_{T}(R,\omega_{m}^{\prime })\delta \left(\omega -\sum_{j=1}^{m}\omega_{j}^{\prime }\right)$$ 11

When computing the fields, it is convenient to explicitly write the linear conductivity contribution to the current and use the fact that the symmetry group (*D*_3*h*_) of monolayer MoS_2_ enforces that second-rank tensors are isotropic, i.e., ${\overset{↔}{\sigma }}^{\left(1\right)}(\omega_{j})=\sigma^{\left(1\right)}(\omega_{j})l$ , where l is the identity operator. The incident field due to a superposition of *N* monochromatic waves is $E_{0}(R,\omega )=2\pi \sum_{\zeta_{0}\in \{\pm \omega_{j}\}}E_{0}^{\zeta_{0}}(R)\delta (\omega -\zeta_{0})$ , where the frequency superscript in $E_{0}^{\omega_{j}}(R)$ indicates that we should take the complex conjugate for negative frequency components of the field. Hence, the transmitted and reflected fields up to leading order in ${\overset{↔}{\sigma }}^{\left(m\right)}$ are given by

$$E_{T}(R,\omega )=2\pi \hbar \sum_{m=1}\sum_{\begin{array}{c} \left(\zeta_{1},\zeta_{2}\cdots \zeta_{m}\right)\\ \in \{\pm \omega_{j}\} \end{array}}T^{\left(m\right)}(\{\zeta_{j}\}):E_{0}^{\zeta_{1}}(R)\cdot \cdot \cdot E_{0}^{\zeta_{m}}(R)\delta \left(\hbar \omega -\sum_{j=1}^{m}\hbar \zeta_{j}\right)$$ 12

$$E_{R}(R,\omega )=2\pi \hbar \sum_{m=1}\sum_{\begin{array}{c} \left(\zeta_{1},\zeta_{2}\cdots \zeta_{m}\right)\\ \in \{\pm \omega_{j}\} \end{array}}R^{\left(m\right)}(\{\zeta_{j}\}):E_{0}^{\zeta_{1}}(R)\cdot \cdot \cdot E_{0}^{\zeta_{m}}(R)\delta \left(\hbar \omega -\sum_{j=1}^{m}\hbar \zeta_{j}\right)$$ 13

where the *m* ^th^ order generalized nonlinear Fresnel transmission and reflection coefficient tensors are

$$T^{\left(m\right)}(\{\zeta_{j}\})=\delta_{m,1}T(\zeta_{1})l-(1-\delta_{m,1})\frac{\mu_{0}cT(\zeta_{1})\times T(\zeta_{2})\cdot \cdot \cdot \times T(\zeta_{m})}{1+n(\omega )+\mu_{0}c\sigma^{\left(1\right)}(\omega )}{\overset{↔}{\sigma }}^{\left(m\right)}(\{\zeta_{j}\})$$ 14

$$R^{\left(m\right)}(\{\zeta_{j}\})=\delta_{m,1}R(\zeta_{1})l-(1-\delta_{m,1})\frac{\mu_{0}cT(\zeta_{1})\times T(\zeta_{2})\cdot \cdot \cdot \times T(\zeta_{m})}{1+n(\omega )+\mu_{0}c\sigma^{\left(1\right)}(\omega )}{\overset{↔}{\sigma }}^{\left(m\right)}(\{\zeta_{j}\})$$ 15

where $\omega =\overset{m}{\underset{j=1}{\sum }}\zeta_{j}$ , $T(\omega )=\frac{2}{1+n(\omega )+\mu_{0}c\sigma^{\left(1\right)}(\omega )}$ , and $R\left(\omega \right)=\frac{1-n\left(\omega \right)-\mu_{0}c\sigma^{\left(1\right)}\left(\omega \right)}{1+n\left(\omega \right)+\mu_{0}c\sigma^{\left(1\right)}\left(\omega \right)}$ are the corresponding linear order coefficients.

When the incident light-fields are pure eigenmodes of the paraxial wave equation, they can be written as $E_{0}^{\omega_{j}}(R)=A_{0}^{n_{j},l_{j}}u_{n_{j},l_{j}}(R)e^{il_{j}\varphi }$ , where $A_{0}^{n_{j},l_{j}}$ is the field amplitude and *n*_*j*_ is the radial index of the orthonormal functions $u_{n_{j},l_{j}}$ ­(*R*). The output electromagnetic fields can be decomposed in an eigenmode superposition as $E_{R,T}(R,\omega )=\sum_{n,l}A_{R,T}^{n,l}u_{n,l}(R)e^{il\varphi }$ . This results in coupling between the pump/seed eigenmodes and conversion/generation of nonlinear output fields with a complex spatial profile. The coupling constant for an *m* ^th^ order process is

$$\Lambda_{\begin{array}{c} n_{1}\cdots n_{m}n_{\mathrm{out}}\\ l_{1}\cdots l_{m}l_{\mathrm{out}} \end{array}}^{\left(m\right)}=2\pi \delta_{l_{\mathrm{out}},\sum_{j=1}^{m}\mathrm{sign}(\omega_{j})l_{j}}\int_{0}^{\infty }Ru_{n_{1},l_{1}}^{\omega_{1}}(R)\cdot \cdot \cdot u_{n_{m},l_{m}}^{\omega_{m}}(R)\times u_{n_{\mathrm{out}},l_{\mathrm{out}}}^{*}(R)dR$$ 16

where the superscript in $u_{n_{j},l_{j}}^{\omega_{j}}$ ­(*R*) denotes that we should take the complex conjugate of the function for negative frequency components of the field. The Kronecker delta in front of the integral expresses the OAM selection rule, as discussed in the main text. Note that, unlike the case of OAM, there is no simple closed form selection rule for the radial index of the eigenmodes $u_{n_{\mathrm{out}},l_{\mathrm{out}}}$ ­(*R*). As the interaction between matter and fields in vdW monolayers takes place at the ultimate limit of dimensionality, the coupling and conversion between spatial modes with distinct radial profiles are enhanced. Indeed, in the case of bulk materials, the intensity distributions of the electromagnetic waves drift away from the direction of the wave vectors as the fields interacts with matter, a phenomenon known as spatial walk-off. This process deteriorates the overlap between the input pump and seed beams, thus leading to a decrease in frequency mixing interactions and mode conversion. On the other hand, 2D systems eliminate the possibility of walk-off and propagation-induced mode mismatch. In the case of second-order processes with two incident beams (pump and seed), the coupling constant that determines the nonlinear output spatial profile reduces to eq 4.

## Supplementary Material



## Notes

Sample characterization and measurement system schematic; SLM phase masks used to generate vortex seed beams; intensity dependence and polarimetry of vortex DFG in monolayer MoS_2_; images, spectra, intensity dependence, and polarimetry of vortex DFG in monolayer WSe_2_; images, spectra, intensity dependence, and polarimetry of vortex SFG in monolayer MoS_2_; images and polarimetry of vortex FWM in monolayer MoS_2_; efficiency of vortex DFG and SFG; theory of polarization of generated nonlinear fields; motivation for the choice of MoS_2_ and WSe_2_; and comment on potential for laser-induced sample damage (PDF)
