# Supplementary material for: Twisted Nonlinear Optics in Monolayer van der Waals Crystals
Source: ACS Nano. 2025 Aug 6;19(34):30919–29. doi: 10.1021/acsnano.5c06908 (PMC12409906; doi:10.1021/acsnano.5c06908)
Supplement: Supplementary file 1 [file nn5c06908_si_001.pdf]

Supporting Information for

Twisted Nonlinear Optics in Monolayer van der Waals Crystals

Tenzin Norden[1,*], Luis M. Martinez[1], Nehan Tarefder[1], Kevin W. C. Kwock[2], Luke M. McClintock[1], Nicholas Olsen[3], Luke N. Holtzman[4], June Ho Yeo[5], Liuyan Zhao[5], Xiaoyang Zhu[3], James C. Hone[6], Jinkyoung Yoo[1], Jian-Xin Zhu[1,7], P. James Schuck[6], Antoinette J. Taylor[1], Rohit P. Prasankumar[1,8], Wilton J. M. Kort-Kamp[7,*], Prashant Padmanabhan[1,*]

[1]Center for Integrated Nanotechnologies, Los Alamos National Laboratory; Los Alamos, NM, 87545, USA

[2]Department of Electrical Engineering, Columbia University; New York, NY, 10027, USA

[3]Department of Chemistry, Columbia University; New York, NY, 10027, USA

[4]Department of Applied Physics and Applied Mathematics, Columbia University; New York, NY 10027, USA

[5]Department of Physics, University of Michigan; Ann Arbor, MI, 48109, USA

[6]Department of Mechanical Engineering, Columbia University; New York, NY, 10027, USA

[7]Theoretical Division, Los Alamos National Laboratory; Los Alamos, NM, 87545, USA

[8]Deep Science Fund, Intellectual Ventures; Bellevue, WA, 98005, USA

*Corresponding authors: tnorden@lanl.gov, kortkamp@lanl.gov, prashpad@lanl.gov

I. Sample Characterization and Measurement System Schematic

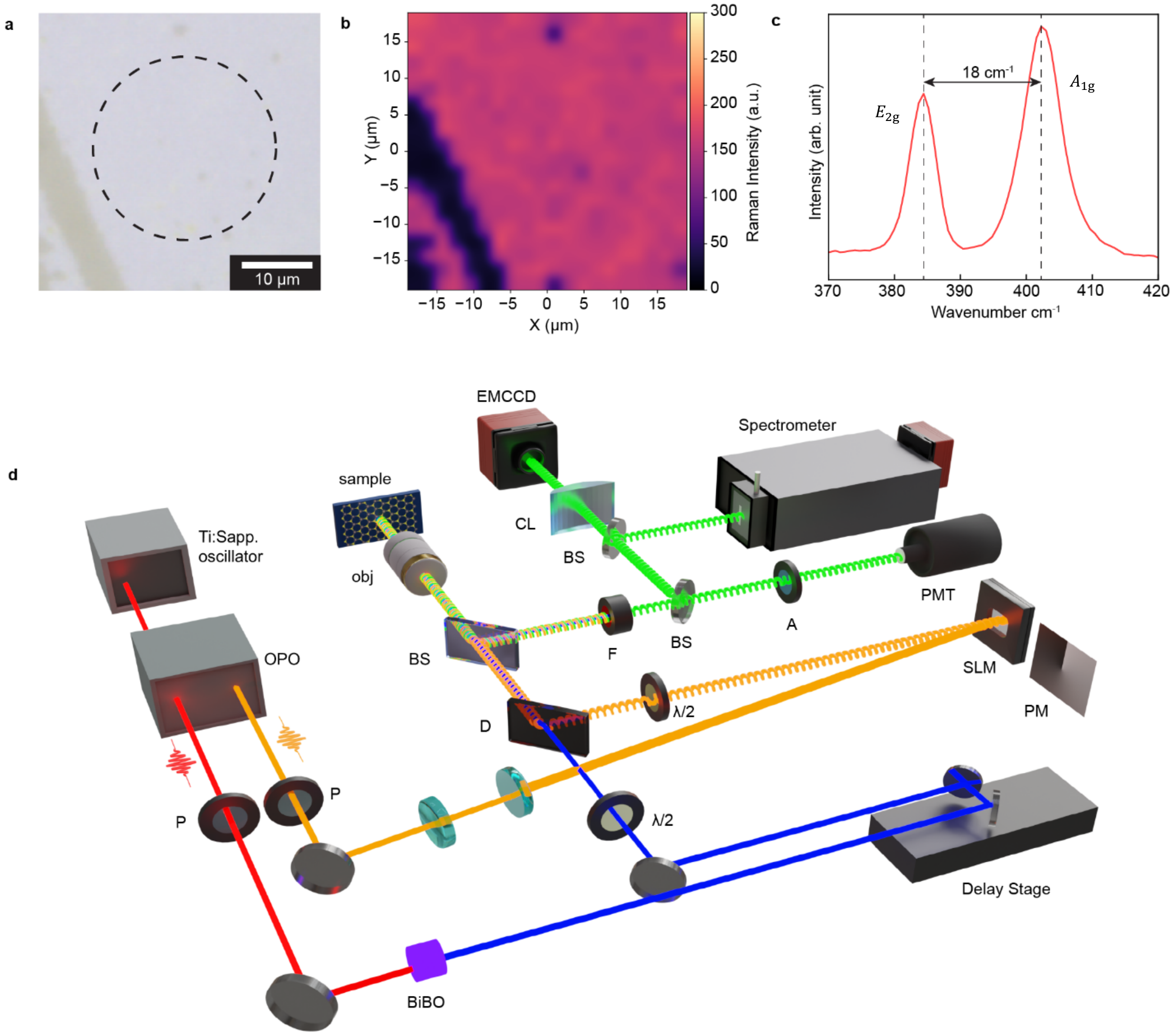

Figure S1. Sample Characterization and experimental system schematic. (a) Microscope image of the monolayer MoS_2 sample on a glass substrate with the circle indicating the maximal size of the photoexcited spot, (b) its post-experiment 2D Raman map tracking the peak intensity of A_{1g} mode under 2.33 eV photoexcitation, and (c) the Raman spectrum under 2.33 eV photoexcitation. (d) Schematic of the time-resolved structured nonlinear optical microscopy system. OPO: optical parametric oscillator, P: polarizer, BiBO: bismuth borate, $\lambda/_2$: half-waveplate, D: dichroic filter, BS: beam splitter, obj: objective, F: filter, PM.: phase mask, SLM: spatial light modulator, A: analyzer, CL: cylindrical lens, PMT: photomultiplier tube, EMCCD: electron-multiplying charge coupled device camera.

II. Spatial Light Modulator Phase Masks Used to Generate Vortex Seed Beams

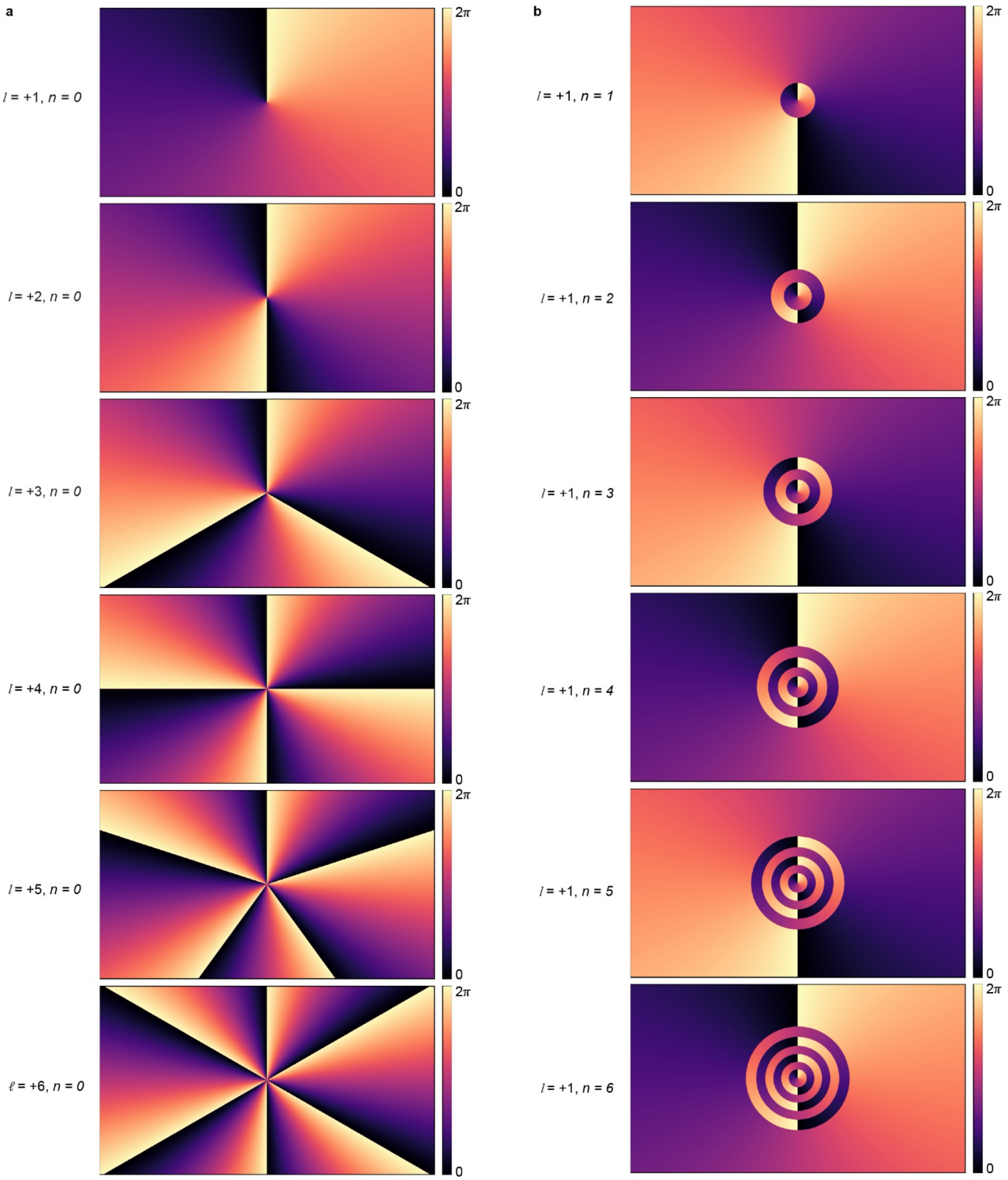

Figure S2. SLM vortex beam phase mask. (a) Phase masks for the generation of vortex beams with topological charges $l = 1 - 6$ and zero radial index ($n = 0$). (b) Phase masks for the generation of $l = +1$ vortex beams with radial index $n = 1 - 6$.

III. Difference Frequency Generation in Monolayer MoS_2

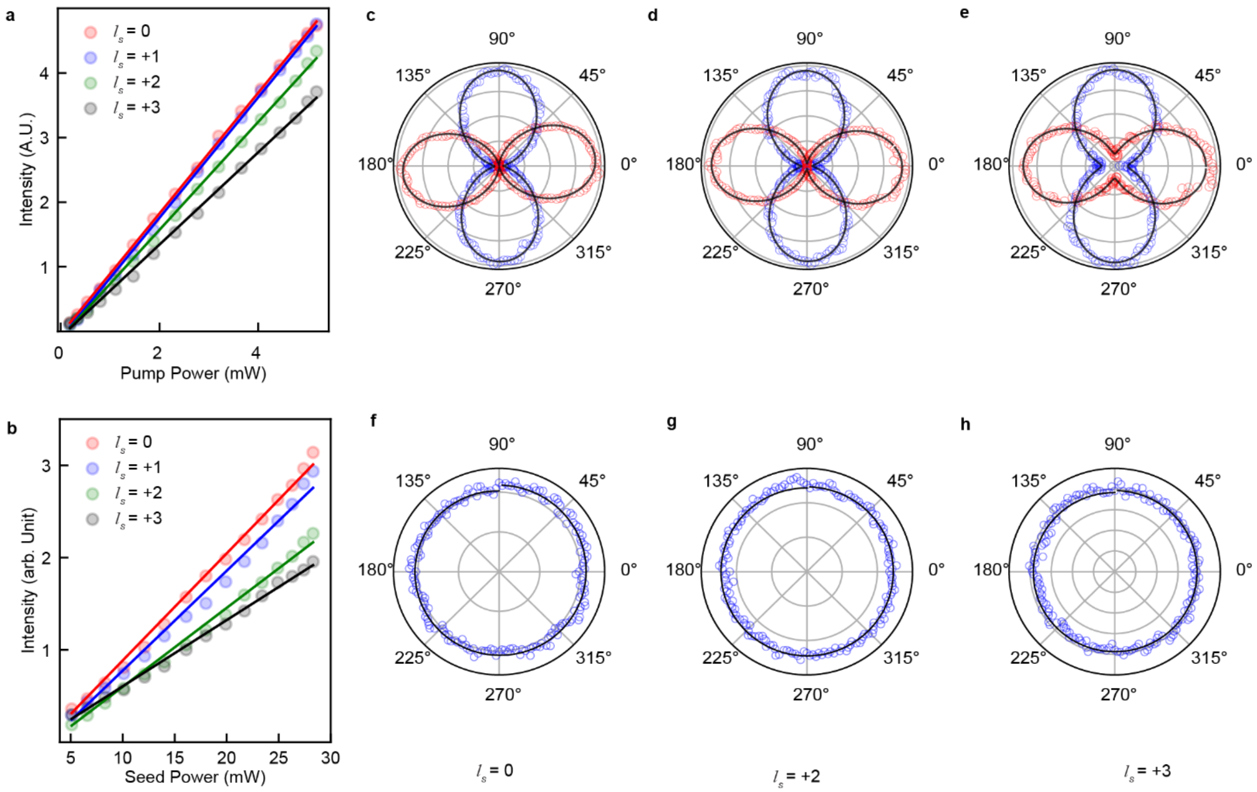

Figure S3. DFG in Monolayer MoS_2. (a-b) DFG output ($\hbar\omega_{DFG} = 1.92$ eV) intensity as a function of (a) pump and (b) seed power for $l_s = 0, +1, +2, +3$ (red, blue, green, and gray circles, respectively). Solid lines are linear fits. (c-e) Polar plots of the DFG output intensity as a function of analyzer angle ($\theta_{DFG} = 0 - 360^{\circ}$) for $l_s = 0, +2, +3$ when the seed beam polarization is parallel to the armchair axis ($\theta_s = 0^{\circ}$) and the pump beam polarization is either perpendicular ($\theta_p = 90^{\circ}$, red pattern) or parallel ($\theta_p = 0^{\circ}$, blue pattern) to the armchair axis. (f-h) Polar plots of the DFG output intensity as a function of pump polarization angle ($\theta_p = 0 - 360^{\circ}$, detected without an analyzer) for $l_s = 0, +2, +3$ and seed polarization fixed parallel to the armchair axis ($\theta_s = 0^{\circ}$). For all the data, the pump beam is Gaussian with energy $\hbar\omega_p^{DFG} = 3.10$ eV and the seed beam energy is $\hbar\omega_s = 1.18$ eV.

IV. Difference Frequency Generation in Monolayer WSe_2

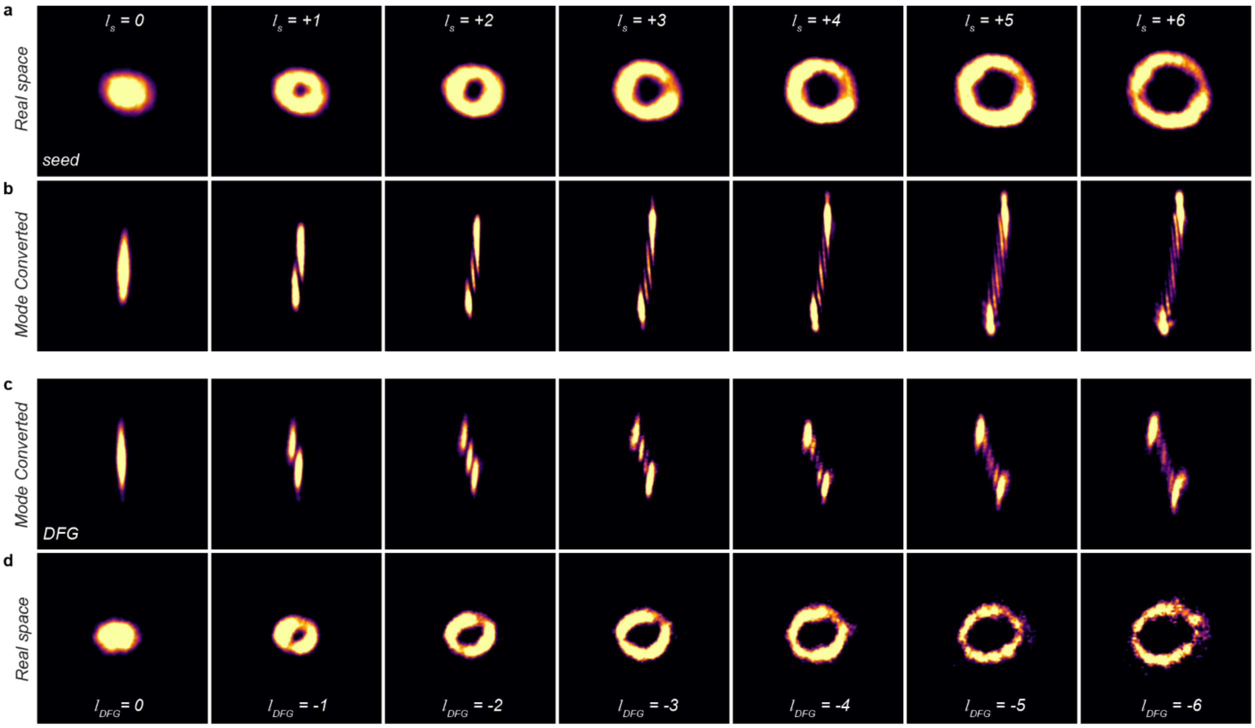

Figure S4. Intensity profile images of DFG in monolayer WSe_2. (a) Intensity profile images of the seed beam ($\hbar\omega_s = 1.18$ eV) and (d) DFG output ($\hbar\omega_{DFG} = 1.92$ eV) for different seed topological charges, $l_s = 0 - 6$. (b-c) mode converted images of the (b) seed and (c) DFG output at the focal plane of a cylindrical lens (f = 120 mm), where $N_F^{DFG} = N_F^s$ and the seed and DFG patterns have opposite skews. For all the data, the pump beams are always Gaussian with $\hbar\omega_p^{DFG} = 3.10$ eV.

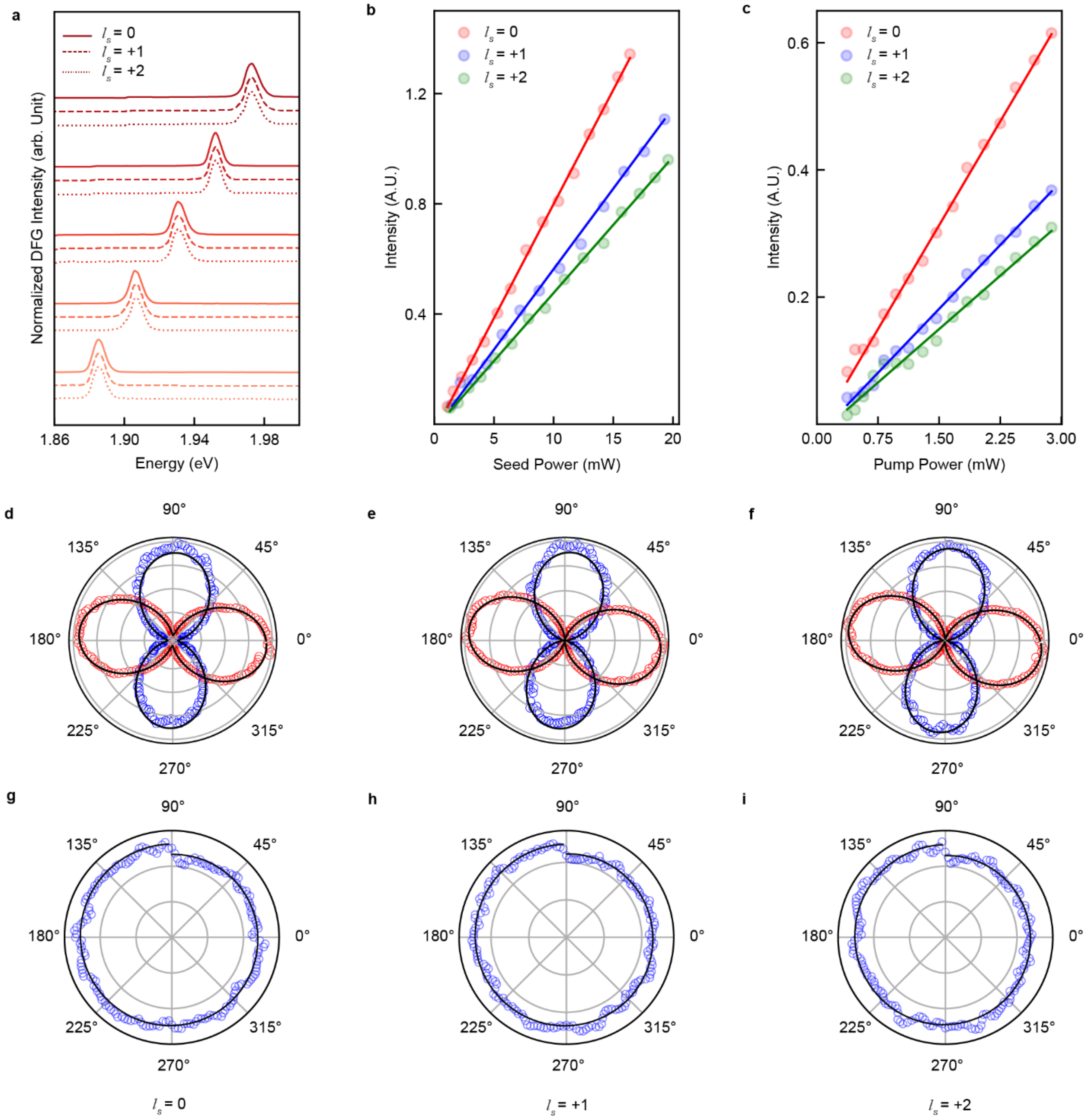

Figure S5. DFG in monolayer WSe₂. (a) Spectra of the DFG output tuned from $\hbar\omega_{DFG}{\sim}1.88 - 1.96$ eV for $l_s = 0, +1, +2$ (solid, dashed, and dotted lines, respectively). (b-c) DFG output ($\hbar\omega_{DFG} = 1.92$ eV) intensity as a function of (b) seed and (c) pump power for $l_s = 0, +1, +2$ (red, blue, and green circles, respectively). Solid lines are linear fits. (d-f) Polar plots of the DFG output intensity as a function of analyzer angle ($\theta_{DFG} = 0 - 360^{\circ}$) for $l_s = 0, +1, +2$ when the seed beam polarization is parallel to the armchair axis ($\theta_s = 0^{\circ}$) and the pump beam polarization is either perpendicular ($\theta_p = 90^{\circ}$, red pattern) or parallel ($\theta_p = 0^{\circ}$, blue pattern) to the armchair axis. (g-i) Polar plots of the DFG output intensity as a function of the pump polarization angle *(*$\theta_p = 0 - 360^{\circ}$, detected without an analyzer*)* for $l_s = 0, +1, +2$ and seed polarization fixed parallel to the armchair axis ($\theta_s = 0^{\circ}$). For the data in (b-i), the pump beam is Gaussian with energy $\hbar\omega_p^{DFG} = 3.10$ eV and the seed beam energy is $\hbar\omega_s = 1.18$ eV.

V. Sum Frequency Generation in Monolayer MoS_2

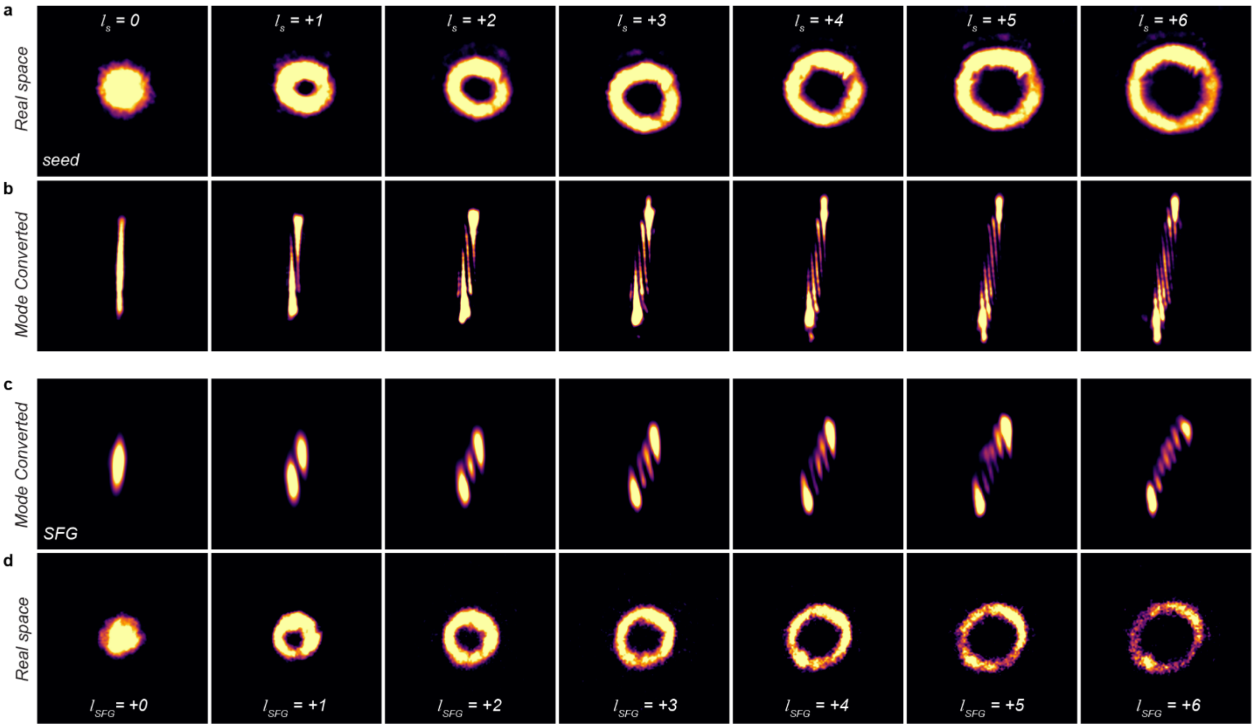

Figure S6. Intensity profile images of SFG in monolayer MoS_2. (a) Intensity profile images of the seed beam ($\hbar\omega_s = 1.18$ eV) and (d) the SFG output ($\hbar\omega_{SFG} = 2.81$ eV) for different seed topological charges, $l_s = 0 - 6$. (b-c) Mode converted images of the (b) seed and (c) SFG output at the focal plane of the cylindrical lens (f = 120 mm), where $N_F^{SFG} = N_F^s$ and the seed and SFG patterns having the same skews. For all data, the pump beams are always Gaussian with $\hbar\omega_p^{SFG} = 1.63$ eV.

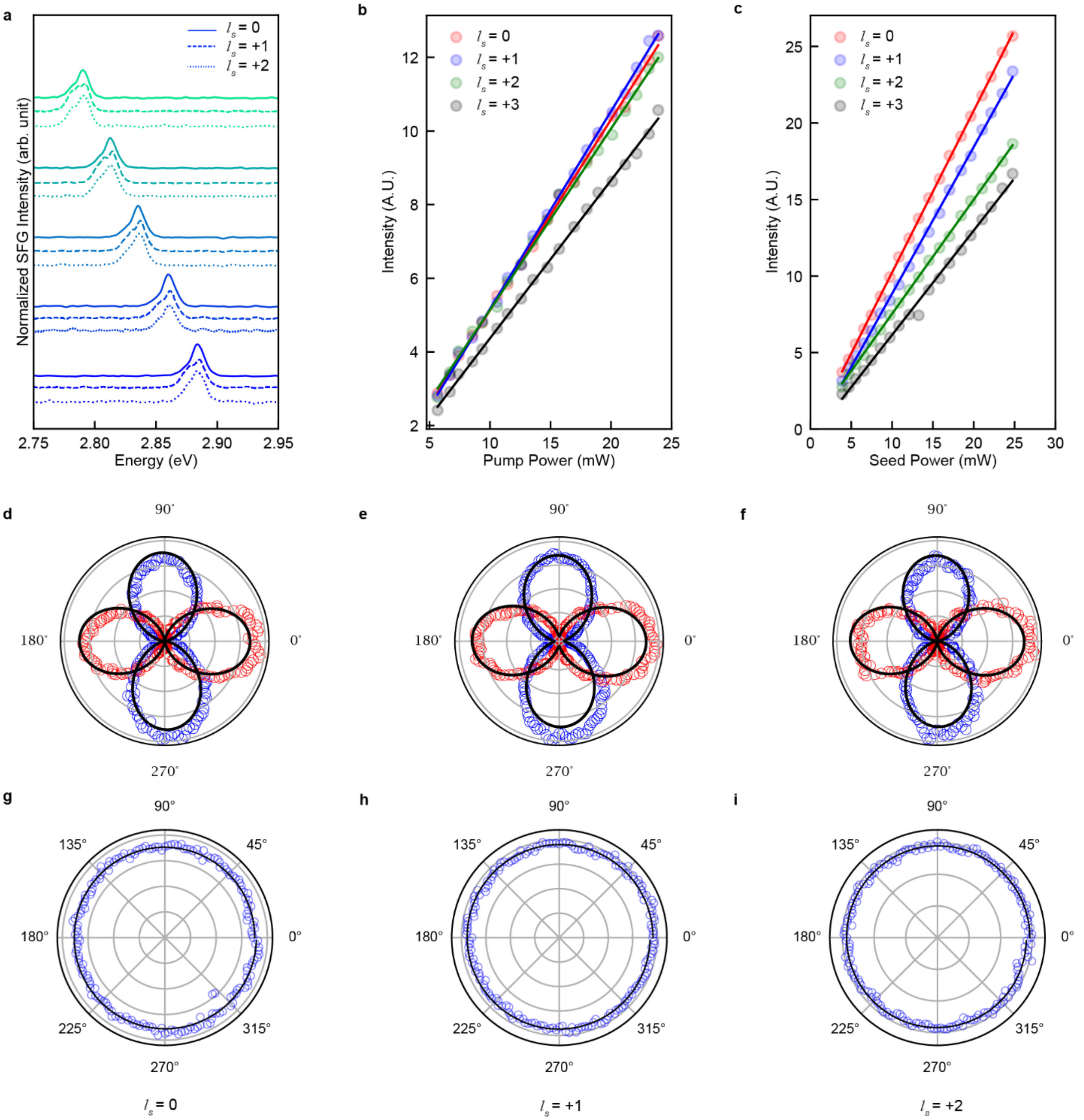

Figure S7. SFG in monolayer MoS₂. (a) Spectra of the SFG output tuned from $\hbar\omega_{SFG}$~$2.78 - 2.87$ eV for $l_s = 0, +1, +2$ (solid, dashed, and dotted lines, respectively). (b-c) SFG output ($\hbar\omega_{SFG} = 2.81$ eV) intensity as a function of the (b) pump and (c) seed power for $l_s = 0, +1, +2, +3$ (red, blue, green, and gray circles, respectively). Solid lines are linear fits. (d-f) Polar plots of the SFG output intensity as a function of analyzer angle ($\theta_{SFG} = 0 - 360^{\circ}$) for $l_s = 0, +1, +2$ when the seed beam polarization is parallel to the armchair axis ($\theta_s = 0^{\circ}$) and the pump beam polarization is either perpendicular ($\theta_p = 90^{\circ}$, red pattern) or parallel ($\theta_p = 0^{\circ}$, blue pattern) to the armchair axis . (g-i) Polar plots of the SFG output intensity as a function of the pump polarization angle ($\theta_p = 0 - 360^{\circ}$, detected without an analyzer) for $l_s = 0, +1, +2$ and seed polarization fixed parallel to the armchair axis ($\theta_s = 0^{\circ}$). For the data in (b-i), the pump beam is Gaussian with energy $\hbar\omega_p^{SFG} = 1.63$ eV and the seed beam energy is $\hbar\omega_s = 1.18$ eV.

VI. Four-Wave Mixing in Monolayer MoS_2

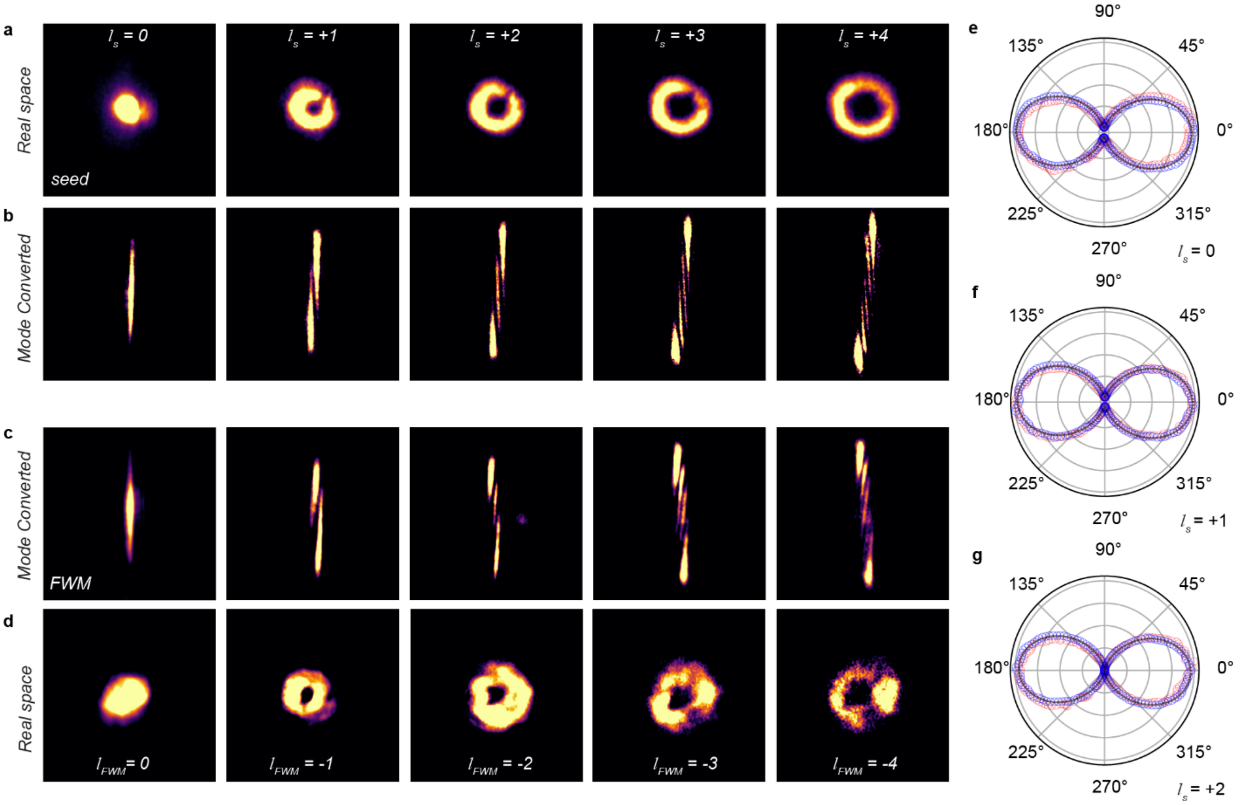

Figure S8. FWM in monolayer MoS_2. (a) Intensity profile images of the seed ($\hbar\omega_s = 1.18$ eV) and (d) the FWM output ($\hbar\omega_{FWM} = 1.90$ eV) for seed topological charges $l_s = 0 - 4$. (b-c) Mode converted images of (b) the seed and (c) the FWM output at the focal plane of the cylindrical lens (f = 120 mm), where $N_F^{FWM} = N_F^s$ and the seed and FWM patterns have opposite skews. (e-g) Polar plots of the FWM output intensity as a function of analyzer angle ($\theta_{FWM} = 0 - 360^{\circ}$) for $l_s = 0, +1, +2$ when the seed polarization is fixed parallel to the armchair axis ($\theta_s = 0^{\circ}$), while the pump polarization is either perpendicular ($\theta_p = 90^{\circ}$, red patterns) or parallel ($\theta_p = 0^{\circ}$, blue patterns) to the armchair axis. For all data, the pump beam is Gaussian with energy $\hbar\omega_p^{FWM} = 1.54$ eV and the vortex seed beam energy is $\hbar\omega_s = 1.18$ eV.

VII. Efficiency of Vortex Difference Frequency Generation and Sum Frequency Generation

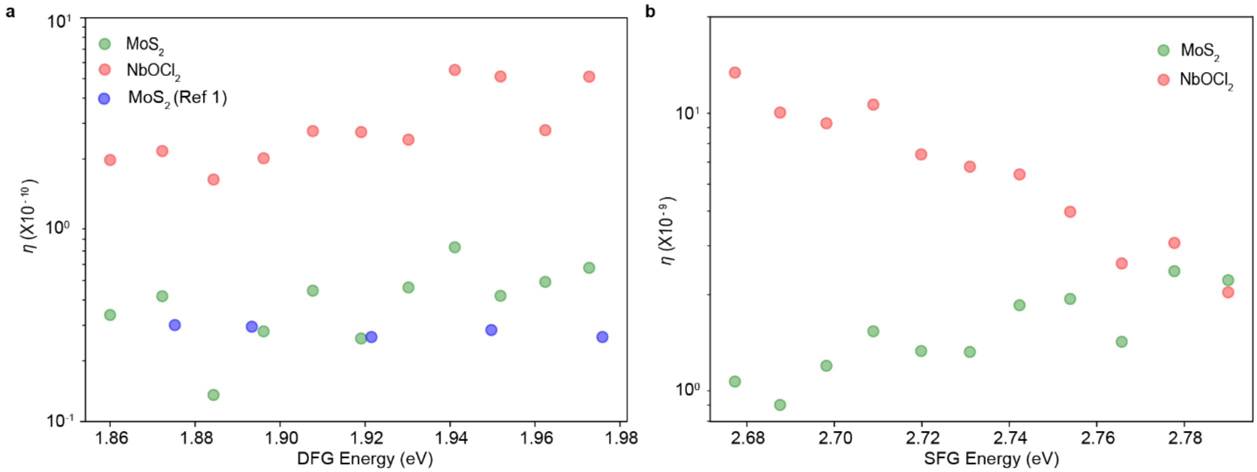

Figure S9. Efficiency (η) measurements with $l_s = +1$. (a) The DFG output efficiencies are plotted as a function of DFG energy for MoS_2 (green circles) and $NbOCl_2$ (red circles). For comparison, the blue circles represent the efficiencies of MoS_2 measured in Ref. 1. (b) The SFG efficiency is plotted as a function of SFG energy for MoS_2 (green circles) and $NbOCl_2$ (red circles).

To extract the absolute DFG and SFG efficiencies, the same backscattering collection geometry utilized in the other studies was used to detect the emitted signals. We assumed that the reflected DFG and SFG are half of the total emitted power ($E_R = E_T$)[1]. The pump and seed fluences were kept constant across all wavelengths for both the DFG and SFG processes under investigation. To measure the power of the emitted DFG (SFG) output, their spectra were captured using a spectrometer equipped with a silicon electron multiplying charge-coupled device (EMCCD) camera. The camera's total digital count output was corrected for all losses from the collection optics before the camera and was then converted to the number of photons. To obtain the counts/photon conversion factor, the normalized photoelectron distribution curve was extracted by correcting for the sensor's gain values and multiplying the DFG (SFG) spectrum by the quantum efficiency (QE) spectrum of the EMCCD sensor. The absolute photoelectron distribution spectrum was then obtained by multiplying the normalized photoelectron distribution curve by the measured photoelectron counts. Finally, the photoelectron distribution spectrum was divided by the QE (wavelength-by-wavelength) to get the total photon distribution spectrum. The sum of each contributing element provided the total incident photon count, from which the efficiencies were extracted. The photon losses from all the collection optics, including the objective lens, beamsplitter, reflecting mirrors, optical filters, focusing lens, and diffraction grating, were compensated for each wavelength using their respective reflection and/or transmission spectrum.

The pump energy for DFG was 3.10 eV (linearly and vertically polarized), while the seed energy was tuned from 1.13 eV to 1.23 eV (also linearly and vertically polarized). The fluences for the pump and seed beams were 35 $\mu J/cm^2$ and 230 $\mu J/cm^2$, respectively. The pump energy for SFG was 1.55 eV (linearly and vertically polarized), while the seed energy was tuned from 1.13 eV to 1.23 eV (also linearly and vertically polarized). The fluences for the pump and seed beams were 80 $\mu J/cm^2$ and 150 $\mu J/cm^2$, respectively.

VIII. Polarization of Generated Nonlinear Fields

We consider that the monolayer transition-metal dichalcogenide is illuminated by two linearly polarized monochromatic electromagnetic waves corresponding to the pump and seed beams. In the paraxial approximation, the characteristic nonlinear response of the monolayer couples only to the polarization degree of freedom of the field. In this scenario, one can determine the local nonlinear polarization generated in the monolayer by expressing the incident fields as

$$\boldsymbol{E_p} = E_p\left(\cos\theta_p\hat{\boldsymbol{x}} + \sin\theta_p\,\hat{\boldsymbol{y}}\right),$$

$$\boldsymbol{E_s} = E_s(\cos\theta_s\hat{\boldsymbol{x}} + \sin\theta_s\,\hat{\boldsymbol{y}}),$$

where $E_{p,s}$ are the position-dependent amplitudes of the fields and $\theta_{p,s}$ are the angles of the electric fields with the armchair direction of the sample. The second and third order polarizations induced on the monolayer are given in terms of the nonlinear susceptibilities as

$$P_i^{(2)}(\omega_m + \omega_n) = \epsilon_0 \sum_{j,k}\sum_{(m,n)} \chi_{ijk}^{(2)}(\omega_m, \omega_n) E_j^{\omega_m} E_k^{\omega_n},$$

$$P_i^{(3)}(\omega_m + \omega_n + \omega_o) = \epsilon_0 \sum_{j,k,l}\sum_{(m,n,o)} \chi_{ijkl}^{(3)}(\omega_m, \omega_n, \omega_o) E_j^{\omega_m} E_k^{\omega_n} E_l^{\omega_o}.$$

The D_{3h} group symmetry of MoS_2 enforces that the only nonzero components of the susceptibility tensor are[2,3]

$$\chi_{yyy}^{(2)} = -\chi_{yxx}^{(2)} = -\chi_{xxy}^{(2)} = \chi_{xyx}^{(2)},$$

and

$$\chi_{xxxx}^{(3)}, \chi_{xyyx}^{(3)}, \chi_{xyxy}^{(3)}, \chi_{xxyy}^{(3)},$$

with $\chi_{xxxx}^{(3)} = \chi_{xyyx}^{(3)} + \chi_{xyxy}^{(3)} + \chi_{xxyy}^{(3)}$. We will neglect frequency dispersion (Kleinman symmetry), in which case the components of the nonlinear polarization simplify to

$$P_x^{(2)}\left(\omega_p \pm \omega_s\right) = -2\epsilon_0\chi_{yyy}^{(2)}\left(E_x^{\omega_p} E_y^{\pm\omega_s} + E_y^{\omega_p} E_x^{\pm\omega_s}\right),$$

$$P_y^{(2)}\left(\omega_p \pm \omega_s\right) = 2\epsilon_0\chi_{yyy}^{(2)}\left(E_y^{\omega_p} E_y^{\pm\omega_s} - E_x^{\omega_p} E_x^{\pm\omega_s}\right),$$

and

$$P_x^{(3)}\left(2\omega_p \pm \omega_n\right) = 2\epsilon_0\chi_{xxxx}^{(3)}\left[\left(3{E_x^{\omega_p}}^2 + {E_y^{\omega_p}}^2\right)E_x^{\pm\omega_s} + 2E_x^{\omega_p}E_y^{\omega_p}E_y^{\pm\omega_s}\right],$$

$$P_y^{(3)}\left(2\omega_p \pm \omega_n\right) = 2\epsilon_0\chi_{xxxx}^{(3)}\left[\left(3{E_y^{\omega_p}}^2 + {E_x^{\omega_p}}^2\right)E_y^{\pm\omega_s} + 2E_y^{\omega_p}E_x^{\omega_p}E_x^{\pm\omega_s}\right].$$

We now consider that the analyzer is polarized along the direction $\hat{\boldsymbol{\eta}} = \cos\varphi\,\hat{\boldsymbol{x}} + \sin\varphi\,\hat{\boldsymbol{y}}$. Hence, the second order field transmitted through the polarizer is given by

$$P_{\hat{\eta}}^{(2)} = P_x^{(2)}\cos\varphi + P_y^{(2)}\sin\varphi = -2\epsilon_0\chi_{yyy}^{(2)}E_pE_s\cos\left(\frac{\pi}{2} - \varphi - \theta_p - \theta_s\right).$$

The argument of the cosine function above defines the relative angle between the analyzer axis and the polarization of the second order electric field, $\theta_{rel} = \theta_{SFG,DFG} - \varphi$. Hence, one concludes that the field due to these second order processes is polarized along the $\theta_{SFG,DFG} = \frac{\pi}{2} - \theta_p - \theta_s$ direction. It is clear from these expressions that the intensity of the field after the analyzer will be proportional to the square of the cosine function, resulting in the bilobed angular distribution reported in Fig. 1 of the main paper. Also, we notice that the angular distribution should rotate by 90° if one keeps the seed (pump) fixed horizontally polarized and rotate the pump (seed) by $\pi/2$ relative to the armchair axis of the crystal.

The case of the third order nonlinear response is similar, but the polarization along the direction of the analyzer axis has a more complicated form

$$P_{\hat{\eta}}^{(3)} = P_x^{(3)}\cos\varphi + P_y^{(3)}\sin\varphi = 2\epsilon_0\chi_{xxxx}^{(3)}{E_p}^2E_s\left[2\cos(\varphi - \theta_s) + \cos\left(\varphi - 2\theta_p + \theta_s\right)\right].$$

This expression can be simplified by noticing that the term in brackets can be cast as $A\cos\beta$, where A and β are real variables that satisfy the following complex equation

$$2e^{i(\varphi-\theta_s)} + e^{i(\varphi-2\theta_p+\theta_s)} = Ae^{i\beta}.$$

The real part of this equation gives the desired simplification for the third order nonlinear polarization, $2\cos(\varphi - \theta_s) + \cos\left(\varphi - 2\theta_p + \theta_s\right) = A\cos\beta$, while the modulus and imaginary parts provide two additional equations that fully determine A and β (up to an arbitrary constant phase factor). Namely, by taking the modulus on both sides of the equation one arrives at

$$A = \sqrt{5 + 4\cos\left(2(\theta_p - \theta_s)\right)},$$

while the imaginary part results in

$$\beta = \varphi - \theta_s + \sin^{-1}\left[\frac{1}{A}\sin\left(2(\theta_p - \theta_s)\right)\right].$$

Like the case of second order nonlinear polarization, here β defines the relative angle between the analyzer axis and the polarization of the third order electric field. Therefore, one obtains that the field due to a four-wave mixing process is polarized along the $\theta_{FWM} = \theta_s - \sin^{-1}\left[\frac{1}{A}\sin\left(2(\theta_p - \theta_s)\right)\right]$ direction. These results imply that the intensity of the field after the analyzer should have a bi-lobed profile proportional to $\cos^2\beta$. One also notices that if the pump and seed beams have the same ($\theta_p = \theta_s$) or orthogonal ($\theta_p = \theta_s \pm \frac{\pi}{2}$) polarizations, the emitted FWM field will share the

same polarization as the seed ($\theta_{FWM} = \theta_s$). These results are consistent with those observed experimentally and reported in Fig. 4 of the main paper.

IX. Motivation for the choice of MoS_2 and WSe_2

Transition metal dichalcogenides (TMDs) such as MoS_2, WS_2, WSe_2, and $MoSe_2$ possess identical point group symmetry and nearly identical monolayer thicknesses. As such, our observation of broadly tunable photon energy, topological charge, and radial index conversion through vortex nonlinear optical processes apply to any of these monolayer systems owing to their shared elimination of both volumetric aberrations (e.g., spatial walk-off) and phase-matching considerations due to their low dimensionality. Our selection of MoS_2 and WSe_2 was, ultimately, motivated only by practical considerations, namely, the ready availability of high-quality single crystals of the former from commercial sources and the latter through the growth capabilities of the co-authors. These bulk crystals enabled us to mechanically exfoliate monolayer flakes of sufficiently large area and uniformity over the length-scale of our focused pump and seed beams (~15 um). This was critical as we were investigating multi-beam mixing of spatially complex light-fields, where inhomogeneity in the monolayer could adversely impact the topological charge or radial index conversion processes under investigation.

X. Comment on potential for laser-induced sample damage

In our experiments, the focal spot diameter of the pump beam was approximately 15 µm, while that of the vortex seed beam varied from ~4 - 12 µm (depending on the value of the seed topological charge, $l = 0 - 6$). This translates to a maximal pump fluence of ~ 0.035 mJ/cm^2 and a maximal seed fluence of ~ 2.78 mJ/cm^2 for the DFG power dependence studies. Both these values are significantly lower than the > 20 mJ/cm^2 damage threshold reported in literature for monolayer MoS_2[4]. It is also important to note that the damage threshold of monolayer TMDs are also highly wavelength-dependent. For example, in our DFG experiments, the pump photon energy ($\hbar\omega_p^{DFG} =$ 3.10 eV) was well above the bandgap of MoS_2 (~1.90 eV), resulting in a higher potential for laser-induced sample damage from the pump due to linear absorption. This is the main reason why we limited our maximum pump power to ~ 5 mW in the power studies. In contrast, the seed beam photon energy ($\hbar\omega_s^{DFG}$ = 1.13 - 1.23 eV) was always well below the bandgap. This results in minimal single-photon absorption and a much lower potential for laser-induced sample damage (which would stem from multi-photon effects). For all our power dependence studies, we observed no visible signs of damage under optical microscopy (conducted periodically during the experiment). Finally, we note that if laser-induced sample degradation had occurred, we would most likely have observed a significant deviation from the expected pump or seed power dependencies. This was not the case in any of our studies, with all power dependencies matching theoretical expectations.

References

(1) Trovatello, C.; Marini, A.; Xu, X.; Lee, C.; Liu, F.; Curreli, N.; Manzoni, C.; Dal Conte, S.; Yao, K.; Ciattoni, A.; Hone, J.; Zhu, X.; Schuck, P. J.; Cerullo, G. Optical Parametric Amplification by Monolayer Transition Metal Dichalcogenides. *Nat. Photonics* **2021**, *15*, 6–10.

(2) Boyd, R. W. *Nonlinear Optics*, 3rd ed.; Academic Press: Amsterdam, 2008.

(3) Shen, Y. R. *The Principles of Nonlinear Optics*; Wiley Interscience: Hoboken, 2002.

(4) Paradisanos, I.; Kymakis, E.; Fotakis, C.; Kioseoglou, G.; Stratakis, E. Intense Femtosecond Photoexcitation of Bulk and Monolayer MoS2. *Appl. Phys. Lett.* **2014**, *105*, 041108.
